# Stepwise connectivity of the entorhinal cortex along connectomic gradients in Alzheimer’s disease

**DOI:** 10.1093/braincomms/fcaf399

**Published:** 2025-10-14

**Authors:** Jazlynn Xiu Min Tan, Min Su Kang, Yi-Hsuan Yeh, Gleb Bezgin, Firoza Z Lussier, Seok-Jun Hong, Jonah Isen, Nesrine Rahmouni, Paolo Vitali, Maxime Montembeault, Jesse M Klostranec, Arthur C Macedo, Tevy Chan, JoAnne McLaurin, Walter Swardfager, Boris C Bernhardt, Bojana Stefanovic, Jean-Paul Soucy, Serge Gauthier, Sandra E Black, Pedro Rosa-Neto, Julie Ottoy, Maged Goubran

**Affiliations:** Department of Medical Biophysics, University of Toronto, Toronto, ON M5G 1L7, Canada; Hurvitz Brain Sciences Program, Sunnybrook Research Institute, University of Toronto, Toronto, ON M4N 3M5, Canada; Hurvitz Brain Sciences Program, Sunnybrook Research Institute, University of Toronto, Toronto, ON M4N 3M5, Canada; Neuroinformatics for Personalized Medicine Lab, Montreal Neurological Institute, McGill University, Montréal, QC H3A 2B4, Canada; Translational Neuroimaging Laboratory, McGill Centre for Studies in Aging, Montreal, QC H4H 1R3, Canada; Center for Neuroscience Imaging Research, Institute for Basic Science, Suwon 86364, Republic of Korea; Hurvitz Brain Sciences Program, Sunnybrook Research Institute, University of Toronto, Toronto, ON M4N 3M5, Canada; Translational Neuroimaging Laboratory, McGill Centre for Studies in Aging, Montreal, QC H4H 1R3, Canada; Translational Neuroimaging Laboratory, McGill Centre for Studies in Aging, Montreal, QC H4H 1R3, Canada; Translational Neuroimaging Laboratory, McGill Centre for Studies in Aging, Montreal, QC H4H 1R3, Canada; Translational Neuroimaging Laboratory, McGill Centre for Studies in Aging, Montreal, QC H4H 1R3, Canada; Translational Neuroimaging Laboratory, McGill Centre for Studies in Aging, Montreal, QC H4H 1R3, Canada; Translational Neuroimaging Laboratory, McGill Centre for Studies in Aging, Montreal, QC H4H 1R3, Canada; Biological Sciences, Sunnybrook Research Institute, Toronto, ON M4N 3M5, Canada; Department of Laboratory Medicine and Pathobiology, University of Toronto, Toronto, ON M5S 1A8, Canada; Hurvitz Brain Sciences Program, Sunnybrook Research Institute, University of Toronto, Toronto, ON M4N 3M5, Canada; Department of Pharmacology and Toxicology, University of Toronto, Toronto, ON M5S 1A8, Canada; McConnell Brain Imaging Centre, Montreal Neurological Institute and Hospital, McGill University, Montreal, QC H3A 2B4, Canada; Department of Medical Biophysics, University of Toronto, Toronto, ON M5G 1L7, Canada; Physical Sciences Platform, Sunnybrook Research Institute, University of Toronto, Toronto, ON M4N 3M5, Canada; McConnell Brain Imaging Centre, Montreal Neurological Institute and Hospital, McGill University, Montreal, QC H3A 2B4, Canada; Translational Neuroimaging Laboratory, McGill Centre for Studies in Aging, Montreal, QC H4H 1R3, Canada; Hurvitz Brain Sciences Program, Sunnybrook Research Institute, University of Toronto, Toronto, ON M4N 3M5, Canada; Division of Neurology, Department of Medicine, University of Toronto, Toronto, ON M5S 3H2, Canada; Translational Neuroimaging Laboratory, McGill Centre for Studies in Aging, Montreal, QC H4H 1R3, Canada; Hurvitz Brain Sciences Program, Sunnybrook Research Institute, University of Toronto, Toronto, ON M4N 3M5, Canada; Physical Sciences Platform, Sunnybrook Research Institute, University of Toronto, Toronto, ON M4N 3M5, Canada; Division of Neurology, Department of Medicine, University of Toronto, Toronto, ON M5S 3H2, Canada; Department of Medical Biophysics, University of Toronto, Toronto, ON M5G 1L7, Canada; Hurvitz Brain Sciences Program, Sunnybrook Research Institute, University of Toronto, Toronto, ON M4N 3M5, Canada; Physical Sciences Platform, Sunnybrook Research Institute, University of Toronto, Toronto, ON M4N 3M5, Canada; Harquail Centre for Neuromodulation, Sunnybrook Health Sciences Centre, Toronto, ON M4N 3M5, Canada

**Keywords:** Alzheimer’s disease, connectome gradients, stepwise connectivity, fMRI, dMRI

## Abstract

The entorhinal cortex is one of the earliest sites of tau tangle deposition in Alzheimer’s disease. Existing connectome studies focus on tau propagation along direct, first-order connections between brain regions, overlooking multi-step, higher-order connections that contribute to the spread of pathology in the brain. We propose a novel quantitative integration of graph theory-based stepwise connectivity with low-dimensional connectome gradient space, which reflects the brain’s hierarchical organization. This allows us to elucidate multi-step connectivity between the entorhinal cortex (seed region) and the rest of the brain along the major axes of functional and structural brain organization. In this study, we included 213 participants from the Translational Biomarkers in Aging and Dementia (103 amyloid-negative cognitively normal, 35 amyloid-positive cognitively normal, and 75 cognitively impaired) with diffusion-weighted MRI, resting-state functional MRI, and 18F-MK6240 tau-PET. Through the novel integration between stepwise connectivity and connectome gradients, we observed hypoconnectivity from the entorhinal cortex to the transmodal end of the functional gradient and to the posterior end of the structural gradient. On the other hand, multi-step connections from the entorhinal cortex showed increased connectivity toward both unimodal (e.g. somatomotor) and transmodal (e.g. frontoparietal) networks of the functional gradient as well as anterior ends of the structural gradient, potentially initiating new paths for tau spread. Finally, tau–connectivity correlations shifted spatially within connectome gradient space, moving from the highest-order (default mode network/limbic) cognitive system of the functional gradient in the preclinical stage (amyloid-positive cognitively normal) to the second-highest order (frontoparietal) system in the clinical stage (cognitively impaired). In conclusion, we demonstrate widespread network reorganization of both direct and indirect, multi-step connections that are associated with patterns of tau spread in Alzheimer’s disease.

## Teaser

Tan *et al*. propose a novel visualization of stepwise connectivity in ‘connectome gradient space’ defined by the principal functional and structural connectomic gradients of the brain. They show widespread network reorganization of both direct and indirect, multi-step connections, associated with patterns of tau spread in Alzheimer’s disease.

## Graphical Abstract

**Graphical Abstract:**
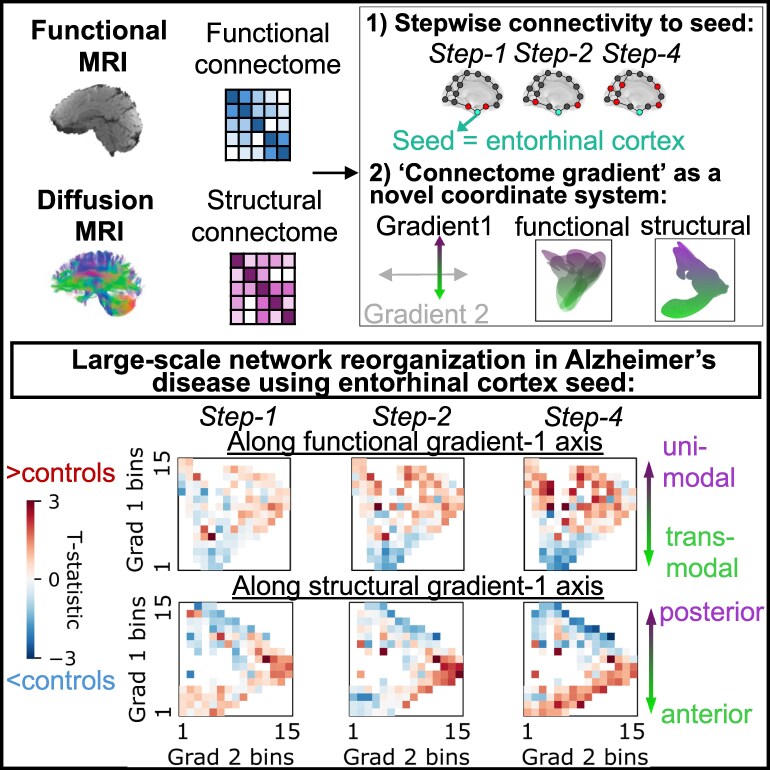

## Introduction

Alzheimer’s disease (AD) is a progressive neurodegenerative disorder characterized by the accumulation of amyloid-β (Aβ) plaques, neurofibrillary tau tangles, and gradual cognitive decline. Evidence suggests that tau accumulates via both structural and functional connections (e.g. trans-neuronal spread or nodal stress models),^1-4^ which is further exacerbated by Aβ.^1^ The entorhinal cortex (EC) is recognized as one of the earliest sites of tau pathology,^5^ before accumulating in limbic regions and the neocortex.^6^ Both direct (one-order neighboring or ‘one-step’) and indirect, multi-step connections to the EC can undergo abnormal or compensatory reorganizations with AD progression, resulting in the characteristic loss of small-worldness topology in AD.^4,7^ As such, understanding the direct and indirect connectivity changes to the EC via neuroimaging could have implications for developing network-targeted interventions that modulate neuronal activity and large-scale connectivity, as well as understanding the effect of network dysfunction on cognitive decline.

Existing connectome studies in AD have predominantly focused on direct (seed-to-target) neural connections between brain regions.^8^ Stepwise functional connectivity (SFC) is a graph theory-based method that additionally considers alternative, indirect connections that pass through higher-order neighboring nodes connecting the seed to the target, via functional connections derived from resting-state functional MRI (fMRI). SFC has been useful in better understanding network-related neurological disorders. For example, in Parkinson’s disease, two disease subtypes (one: tremor-dominant and two: postural-instability gait disorder) showed altered SFC in gait-related regions. However, the more severe second subtype had higher cerebellar SFC compared with the first subtype, which supports the hypothesis of a compensatory mechanism to counteract the more severe pathology.^9^ SFC can therefore be useful in understanding the progression of neurodegenerative diseases. To our knowledge, only two other studies have investigated stepwise connectivity in AD. Sepulcre *et al*.^10^ used amyloid maps and functional connectivity maps derived from Positron Emission Tomography (PET) and fMRI, respectively, and used the hippocampal formation and parahippocampal gyrus as temporal seed regions. Costumero *et al*.^11^ instead used the optimal connectivity distance, which is the step at which SFC (calculated from functional connectomes) between two nodes is maximized. This enabled insight into the increased functional network separation of the default mode network (DMN), frontoparietal, and salience networks from the rest of the brain in AD, but did not consider in detail how changes to direct and indirect, multi-step connections were associated with this network separation or tau burden.

SFC analysis has been previously studied in conjunction with connectome gradients,^12^ an emerging method to characterize the hierarchical organization of the brain. Connectome gradients capture the gradually shifting and overlapping modes of connectivity variation in the brain, revealing the main principles of large-scale organization of brain function/structure and its association with cognition.^13,14^ For example, gradient 1 (explaining most variance) based on resting-state fMRI uniquely captures the smooth spatial transitions in connectivity variation from primary sensory and motor cortices to the association systems implicated in more integrative cognitive processes.^12^ Dong *et al*.^15^ previously showed that both functional connectome gradients and SFC values of regions involved in higher-order cognitive functions correlated with clinical severity scores of Schizophrenia. Hong *et al*.^16^ proposed a closer qualitative integration of gradients and SFC analysis by visualizing the trajectory of SFC in functional connectome gradient space. They showed that, in controls, SFC seeded from unimodal sensory cortices converged in the transmodal DMN, but this convergence did not occur in patients with autism spectrum disorder. This integrated network analysis was made possible by assessing indirect, multi-step connections via SFC analysis. The gradient space can serve as a novel low-dimensional coordinate space to assess SFC-based direct (‘step one’) and indirect (‘steps > one’) connectivity to key brain hubs or pathological epicenters like the EC. To our knowledge, our study is the first application of both stepwise connectivity to structural connectomes (stepwise structural connectivity or SSC) and SFC in AD. We also perform novel quantitative integration of SFC/SSC and connectome gradients to characterize the topological (re-)organization in AD.^15,16^ This allows the brain-wide identification of key direct and indirect interactions between pathological (tau) epicenters and the rest of the brain. We hypothesize that functional and structural imaging-derived connections (intra-/inter-network) to the EC will undergo reorganization in AD, with hypoconnectivity within temporo-occipital and DMN networks as well as inter-network connectivity changes to other brain networks, including the fronto-parietal network. We further hypothesize that these changes will correlate with tau deposition at a later stage in the disease.

## Materials and methods

### Subjects

We employed the Translational Biomarkers in Aging and Dementia (TRIAD) cohort in this study. The TRIAD cohort aims to characterize the biomarker drivers of dementia. All subjects underwent T1-weighted MRI, diffusion-weighted MRI, resting-state fMRI, ^18^F-MK6240 tau-PET, ^18^F-NAV4694 Aβ-PET, and an extensive neuropsychological battery. Our sample consisted of 213 subjects, of which 103 were cognitively normal Aβ-negative (A− CN), 35 cognitively normal Aβ-positive individuals (A+ CN), and 75 cognitively impaired Aβ-positive (CI) individuals with mild cognitive impairment or AD dementia.^17^ Demographic data are described in Table 1. Data processing was as described in Ottoy *et al*.^4^ In brief, resting-state fMRI data was acquired with single-shot full k-space multiband echo-planar imaging (TR = 0.681 s, TE = 32 ms, slice thickness = 2.5 mm, number of slices = 54, flip angle = 50 degrees, number of measurements = 870, matrix size: 88 × 88, voxel size = 2.5 mm^3^ isotropic, and eyes open fixed on a cross). Slicing timing correction, skull stripping, and registration to MNI152 space were performed using fMRIPrep v.20.2.3. Confound regression using the CompCor method was carried out in Nilearn using the *fmriprep.load_confounds* function. All fMRI to T1 and T1 to atlas registrations were visually checked. One A− CN subject had to be excluded due to motion artifacts. Diffusion-weighted MRI was collected using EPI (TR = 3500 ms, TE = 71 ms, flip angle = 90 degrees, field of view = 232 × 232 × 162, voxel size = 2 mm^3^ isotropic, and 13, 48, and 60 isotropically distributed diffusion-sensitizing gradients with b-value = 0, 1000, and 2000 s/mm^2^, respectively, as well as five b0 images). Susceptibility distortions, motion, Gibbs ringing, and eddy currents were corrected for in FSL v6.0.5 and MRtrix3 v3.0.3. During slice-to-volume motion correction with FSL Eddy, outlier frames, defined as where >20% of the slices within the frame were detected as outliers, were removed.

**Table 1:** Demographics

		Overall	A− CN	A+ CN	CI	*P*-value*
		*n* = 213	*n* = 103	*n* = 35	*n* = 75	
Diagnosis, *n* (%)	CN	138 (64.8)	103 (100.0)	35 (100.0)		<0.001
	MCI	41 (19.2)			41 (54.7)	
	AD	34 (16.0)			34 (45.3)	
Sex, *n* (%)	F	129 (60.6)	63 (61.2)	24 (68.6)	42 (56.0)	0.447
	M	84 (39.4)	40 (38.8)	11 (31.4)	33 (44.0)	
Age, mean (SD)		69.4 (9.2)	68.7 (9.7)	73.5 (7.8)	68.3 (8.8)	0.012^a^
Education, mean (SD)		15.3 (3.8)	15.8 (4.1)	14.6 (3.6)	15.1 (3.5)	0.219
APOE-e4, *n* (%)	Non-carriers	132 (63.2)	75 (73.5)	26 (74.3)	31 (43.1)	<0.001
	Carriers	77 (36.8)	27 (26.5)	9 (25.7)	41 (56.9)	
MMSE, mean (SD)		27.4 (4.2)	29.1 (1.2)	28.9 (1.2)	24.3 (5.8)	<0.001^b^
CDR, mean (SD)		0.2 (0.4)	0.0 (0.1)	0.0 (0.1)	0.7 (0.5)	<0.001
Amyloid SUVR, mean (SD)		1.7 (0.5)	1.2 (0.1)	1.8 (0.3)	2.2 (0.4)	<0.001

AD, Alzheimer’s disease; CI, cognitively impaired A+; CN, cognitively normal; MCI, mild cognitive impairment. *Based on ANOVA with Bonferroni correction and Tukey’s *post hoc* testing. ^a^A− CN or CI versus A+ CN: *P* = 0.02. ^b^A− CN or A+ CN versus CI A+: *P* ≤ 0.001.

### Connectome and gradient extraction

Subject-specific functional and structural connectomes were estimated using regional time series correlations^18^ and the number of reconstructed cross-sectional streamlines via probabilistic fibre tractography,^19^ respectively, using an in-house high-resolution atlas adapted from Glasser *et al*.^4,20^ The functional connectome was Fisher R-to-Z transformed, while weak and spurious correlations were removed by thresholding for the top 20% of connections (as in our previous work^4^) for each region-of-interest (ROI) to achieve fixed edge density.^11^ Diffusion embedding with a cosine kernel was applied to non-linearly reduce the group-averaged connectivity matrices to orthogonal components (gradients) via BrainSpace (http://github.com/MICA-MNI/BrainSpace), which formed a low-dimensional embedding space per diagnostic group.^12,21^ The parameters *α* = 0.5 and *t* = 0 preserve global relations between data points in embedded space during diffusion embedding. Procrustes alignment was performed on each subject’s gradient to realign it to their corresponding group-wise (template) gradient derived from the group-averaged functional or structural connectivity matrix.^21^

### Stepwise connectivity

**Figure 1:**
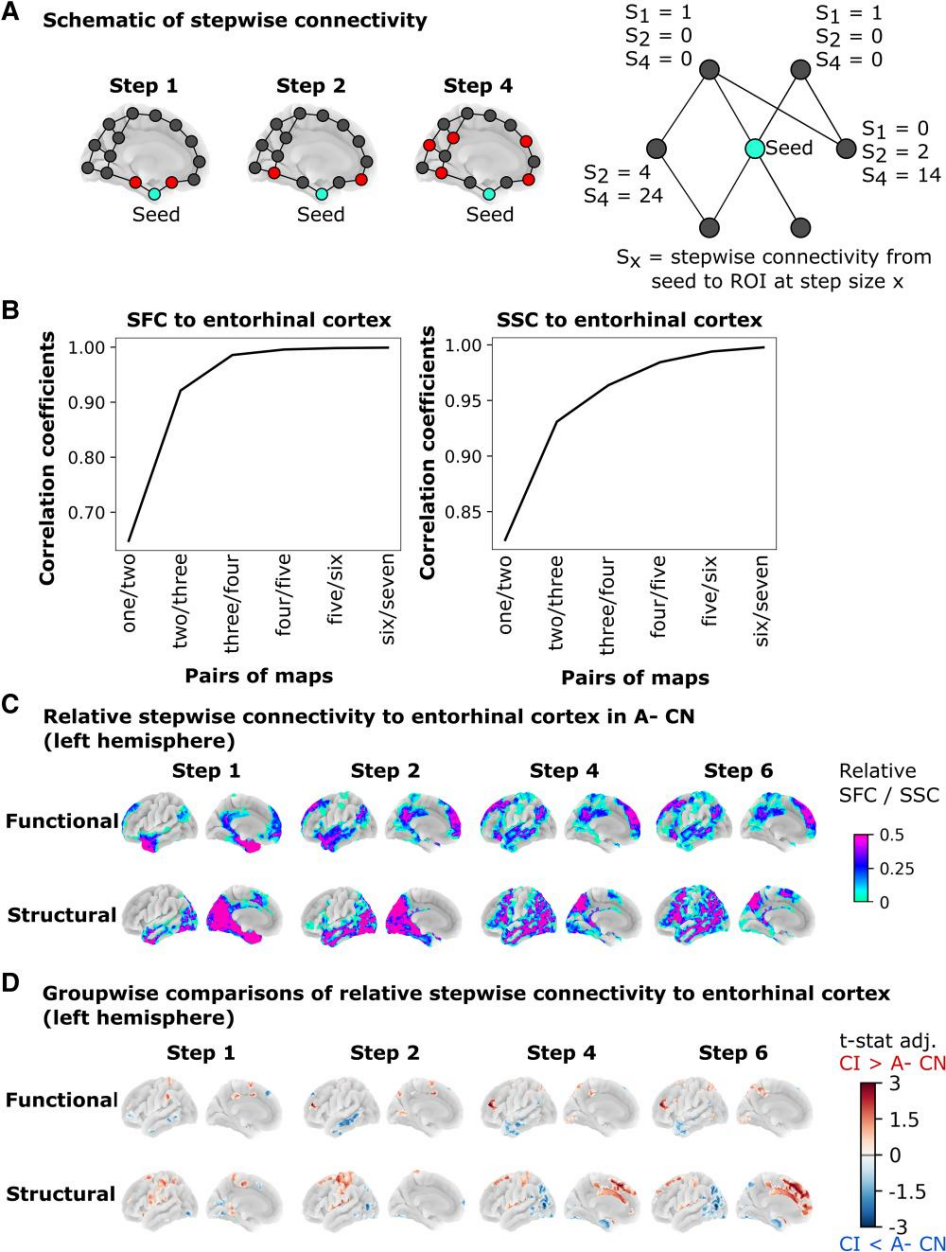
**Whole-brain stepwise connectivity to the EC seed.** (**A**) The number of walks leading from the seed to the target ROI for step size × forms the stepwise connectivity value (S_x_) of that target ROI. (**B**) To check for topological stability of our results, we performed spatial correlation (ROI-wise Pearson R) between consecutive pairs of SFC/SSC maps averaged across the A− CN group (e.g. correlation of step-size 1 with 2, step-size 2 with 3, etc). Convergence was observed at step 7 for both SFC and SSC. (**C**) In A− CN (*N* = 103), SFC propagated from the EC seed to DMN and medial/anterior temporal lobes, while SSC moved from caudal to rostral cortices. The color scale corresponds to within-subject normalized SFC/SSC to the mean and standard deviation across all ROIs (per step size and averaged across the A− CN group) per ROI. (**D**) Groupwise comparisons via a linear regression of within-subject normalized stepwise connectivity showed both reduced and increased SFC/SSC trajectories across step sizes in CI (*N* = 75) compared with A− CN. Target ROIs with increased SFC/SSC in CI compared with A− CN are shown in red, while target ROIs with reduced SFC/SSC in CI compared with A− CN are shown in blue. Only target ROIs with significant *P*-values after adjustment are shown. Results are shown for the left hemisphere (see Supplementary Figs. 1C and 2A, B for right hemisphere results). CN, cognitively normal; CI, cognitively impaired; ROI, region-of-interest; SFC, stepwise functional connectivity; SSC, stepwise structural connectivity.

In a graph representation of the connectome, an edge refers to the (functional or structural) connection between two nodes. A walk in the graph involves passing along these edges from node to node. For example, a walk with an edge length of one goes from a node to its neighbor, while a walk with an edge length of three will pass along three edges to arrive at the target node. The SFC or SCC value assigned to an ROI denotes the number of walks of a particular edge length (one to N edges) that connect the seed (EC) to that ROI^16,22^ (Fig. 1A). SFC/SSC values converged at step size seven (Fig. 1B), in line with other SFC studies.^11,22,23^ As the degree of SFC exponentially grows with increasing steps, we standardized the value at each step size by subtracting the mean value across all ROIs for that subject and dividing by their standard deviation, as per previous work^16^; we refer to these values as within-subject normalized SFC/SSC.

### PET imaging

Standardized uptake value ratio (SUVR) maps were normalized to the inferior portion of the cerebellar grey matter. Aβ-positivity status was determined based on the visual rating of the Aβ-PET images, with the final rating based on a consensus of two physicians who specialize in dementia imaging.

### Statistical analysis

First, we performed group-wise comparisons between SFC/SSC values (normalized across ROIs or non-normalized in Supplementary material) via linear regression adjusted for age, sex, and APOE-ε4 status. T-statistics maps were adjusted for family-wise errors for multiple comparisons with a false-positive rate at *P* < 0.01 and a cluster-wise threshold of 500 voxels. Second, we investigated these group-wise SFC/SSC trajectories within a gradient coordinate system spanned by the two connectivity gradients that explained the most pseudo-variance in the data (functional gradients one-two: 71%, structural gradients one-two: 48%). To this end, we created a heatmap of the groupwise mean of within-subject normalized SFC/SSC and a heatmap of the t-statistic at each step, with ROIs placed at coordinates corresponding to their gradient values in their respective group-averaged template space. In addition, we summed the normalized SFC/SSC across steps and performed groupwise comparisons via linear regression adjusted for age, sex, and APOE-ε4 status, visualizing the *t*-statistic as a heatmap. For each ROI, linear regression of baseline tau against normalized SFC/SSC (the difference between summed SFC/SSC and the A− CN group average) was performed, adjusting for age, sex, and APOE-ε4 status. For analyses related to the sum of normalized SFC/SSC across steps and association with baseline tau, one subject in the control group (*N* = 102) was dropped due to missing baseline tau measurements. For all heatmaps, we smoothed them for visualization by allocating ROIs into 15 equally-sized bins along each axis and displaying the mean of each bin.

## Results

### AD affects both direct and indirect, multi-step functional and structural connections to the EC

We focused on the EC seed as a key epicenter in tau propagation and one of the earliest sites of tau accumulation in typical AD.^1,24^ Among the A− CN group, SFC was highest in the medial-temporal network (step one, closest to EC seed) and propagated to regions of the DMN at step 2 before shifting to sensorimotor regions at steps three to seven. SSC from the EC propagated from posterior (steps one—two) to anterior (steps three—seven) regions (Fig. 1C).

The SFC/SCC maps were visually similar between diagnostic groups (maps were within-subject normalized across ROIs to indicate stepwise changes relative to the rest of the brain) (Supplementary Fig. 1A and B). However, certain networks were reached faster or slower depending on the diagnostic group considered. In CI compared with A− CN, we observed progressively reduced connectivity with increasing step size, that is, reduced SFC from the left EC to medial-temporal network (e.g. middle temporal gyrus at step five: *t* = −3.79, *Padj* < 0.01) and DMN (e.g. middle temporal gyrus at step five: *t* = −3.79, *Padj* < 0.01), as well as reduced SSC between the left EC and visual regions (e.g. visual area V4 at step six: *t* = −5.29, *Padj* < 0.01). Similarly, there was progressively increased SFC from the left EC to frontoparietal and sensorimotor regions and increased SSC to the sensorimotor and medial-frontal/anterior regions in CI compared with A− CN (Fig. 1C and D). In line with traditional connectome analyses, the non-normalized SFC/SSC maps at step one showed primarily hypoconnectivity in DMN/limbic regions in CI compared with A− CN (Supplementary Fig. 2B). We did not observe regions with significant differences between A+ CN and A− CN after FWER correction (Supplementary Fig. 2C).

As the EC is a relatively small region, we repeated analyses with the posterior inferotemporal region in the Glasser atlas. Similar results to the EC were observed for the SFC (Supplementary Fig. 3) and SSC from the posterior inferotemporal seed (Supplementary Fig. 4).

### Integration of gradients with stepwise connectivity reveals large-scale network reorganization in AD along the major axes of brain organization

As gradients capture spatial patterns of connectivity in an easily interpretable low-dimensional space, we next sought to situate the within-subject normalized SFC/SSC alterations in AD in connectome gradient space. To this end, we created a heatmap of the groupwise mean of the SFC/SSC and a heatmap of their group comparisons (*t*-statistic) at each step within connectome gradient space.

**Figure 2:**
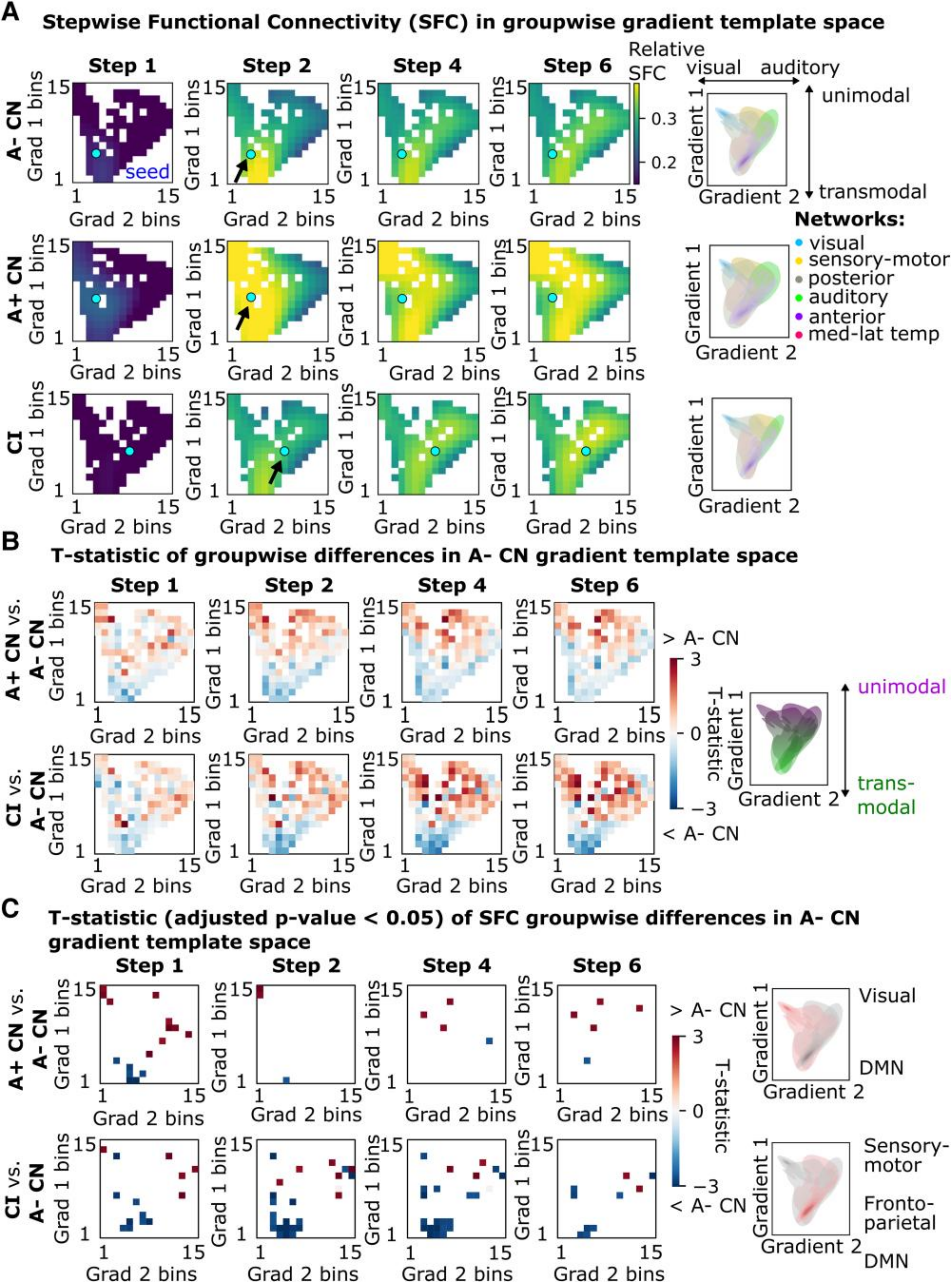
**Stepwise functional connectivity (SFC) visualized along the axes of the gradients of the brain.** (**A**) Within-subject normalized (across ROIs) SFC to the EC projected in the template functional gradient space for each group (A− CN: *N* = 103, A+ CN: *N* = 35, CI: *N* = 75). SFC values in ROIs are smoothed into 15 bins along each gradient for visualization (see Supplementary Fig. 5 for the unsmoothed version). Cyan marker and black arrow indicate the location of the EC seed. Density plots of target ROIs are colored by major networks projected in the template functional gradient space of each group (right). Both gradients have been min-max scaled to range from 0 to 1 to allow direct comparison between groups (see Supplementary Fig. 10 for the unscaled version). (**B**) ROI-wise SFC group comparisons via a linear regression revealed reduced and increased SFC from the EC to transmodal and unimodal networks, respectively, in CI (*N* = 75) compared with A− CN (*N* = 103). In A+ CN (*N* = 35) compared with A− CN, there was widespread increased SFC across the gradient space. Right panel highlights the unimodal-transmodal gradient. (**C**) T-statistics surviving correction for family-wise errors where the adjusted *P*-value < 0.05. Color scale indicates *t*-statistic. Key networks and subnetworks are highlighted in the right panel. Red pixels indicate increased SFC in A+ CN/CI compared with A− CN, while blue pixels indicate reduced SFC in A+ CN/CI compared with A− CN. CN, cognitively normal; CI, cognitively impaired; EC, entorhinal cortex; ROI, region-of-interest; SFC, stepwise functional connectivity.

The first *functional* gradient describes the ‘transmodal-unimodal’ axis of the brain, while the second functional gradient describes the ‘auditory-visual’ axis. On average, A+ CN showed trends of accelerated SFC propagation across steps from the EC to the rest of the brain (Fig. 2A, yellow pixels). Compared with A− CN, CI showed diminished SFC to the DMN at the transmodal pole of gradient one (Fig. 2B and C: blue *t*-stats, step 2: *t* = −3.7 DMN, step 4: *t* = −3.4 DMN, Padj < 0.01), while accelerated propagation (across steps) to the non-DMN regions at the transmodal pole and to the sensorimotor regions at the unimodal pole of gradient one (Fig. 2A: yellow pixels, Fig. 2B, C: red *t*-stats, step 2: *t* = +3.6 sensorimotor, step 2: *t* = +3.5 frontoparietal network, step 2: *t* = +2.9 cingulo-opercular network, step 4: *t* = +3.1 frontoparietal network, step 4: *t* = +2.9 dorsal attention network & cingulo-opercular network, Padj< 0.01), which were not revealed by standard SFC analysis (anatomical space, Fig. 1), (see Supplementary Fig. 5 for unsmoothed heatmaps). In the right hemisphere, there was increased SFC across the gradient space (Supplementary Fig. 6), which was not apparent in anatomical space comparisons (Supplementary Fig. 2A). This may suggest that the right EC remains more functionally connected to the right neocortex in comparison to the left EC to the left neocortex.

**Figure 3:**
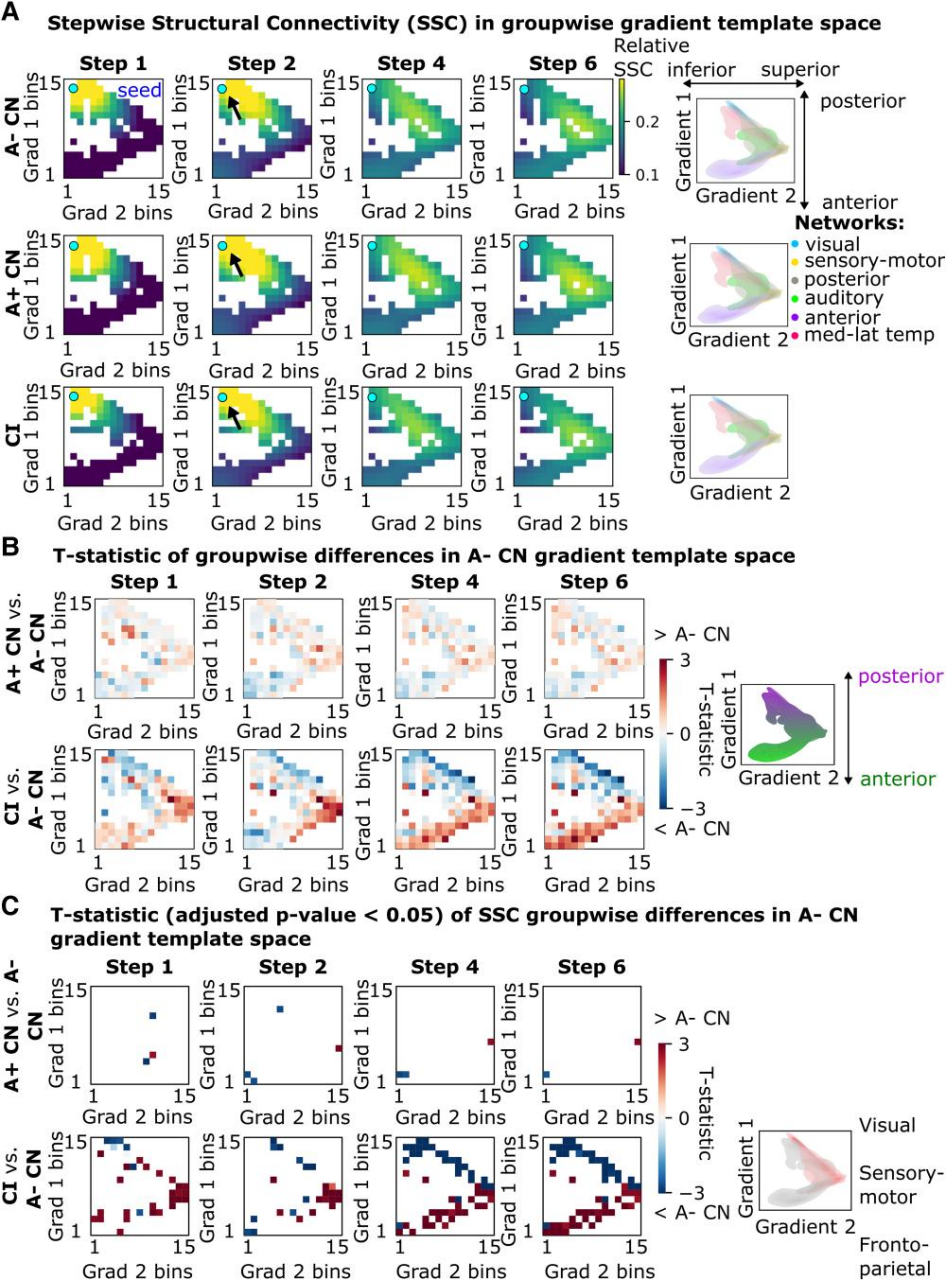
**Stepwise structural connectivity (SSC) visualized along the axes of the gradients of the brain.** (**A**) Within-subject normalized (across ROIs) SSC to the EC projected in the template structural gradient space for each group (A− CN: *N* = 103, A+ CN: *N* = 35, CI: *N* = 75). SSC values in ROIs are smoothed into 15 bins along each gradient for visualization (see Supplementary Fig. 7 for the unsmoothed version). Cyan marker and black arrow indicate the location of the EC seed. Density plots of target ROIs are colored by major networks projected in the template functional gradient space of each group (right). Both gradients have been min-max scaled to range from 0 to 1 to allow direct comparison between groups (see Supplementary Fig. 10 for the unscaled version). (**B**) ROI-wise SSC group comparisons via a linear regression revealed reduced SSC from the EC to temporal-posterior regions in CI (*N* = 75) compared with A− CN (*N* = 103). In A+ CN (*N* = 35) compared with A− CN, there was increased SSC from the EC to the temporal-posterior regions. Right panel highlights the posterior-anterior gradient. (**C**) T-statistics surviving correction for family-wise errors where the adjusted *P*-value < 0.05. Color scale indicates *t*-statistic. Key networks and subnetworks are highlighted in the right panel. Red pixels indicate increased SSC in A+ CN/CI compared with A− CN, while blue pixels indicate reduced SSC in A+ CN/CI compared with A− CN. CN, cognitively normal; CI, cognitively impaired; EC, entorhinal cortex; ROI, region-of-interest; SSC, stepwise structural connectivity.

The first *structural* gradient describes the ‘anterior-posterior’ axis of the brain, while the second structural gradient describes the ‘superior-inferior’ axis. SSC was diminished at the posterior pole (Fig. 3A–C: blue pixels, step 2: *t* = −4.5 visual, step 4: *t* = −5.1 visual, Padj < 0.01) and elevated at the anterior pole in CI compared with A− CN (Fig. 3A–C: red pixels, step 2: *t* = +6 cingulo-opercular network, step 2: *t* = +4.2 sensorimotor, step 4: *t* = +4.6 frontoparietal network, step 4: *t* = +4.3 cingulo-opercular network, Padj < 0.01). See Supplementary Fig. 7 for unsmoothed heatmaps. Right hemisphere results (Supplementary Fig. 8) were similar to those of the left hemisphere.

### The association between tau and connectivity shifts within gradient space with disease progression

**Figure 4:**
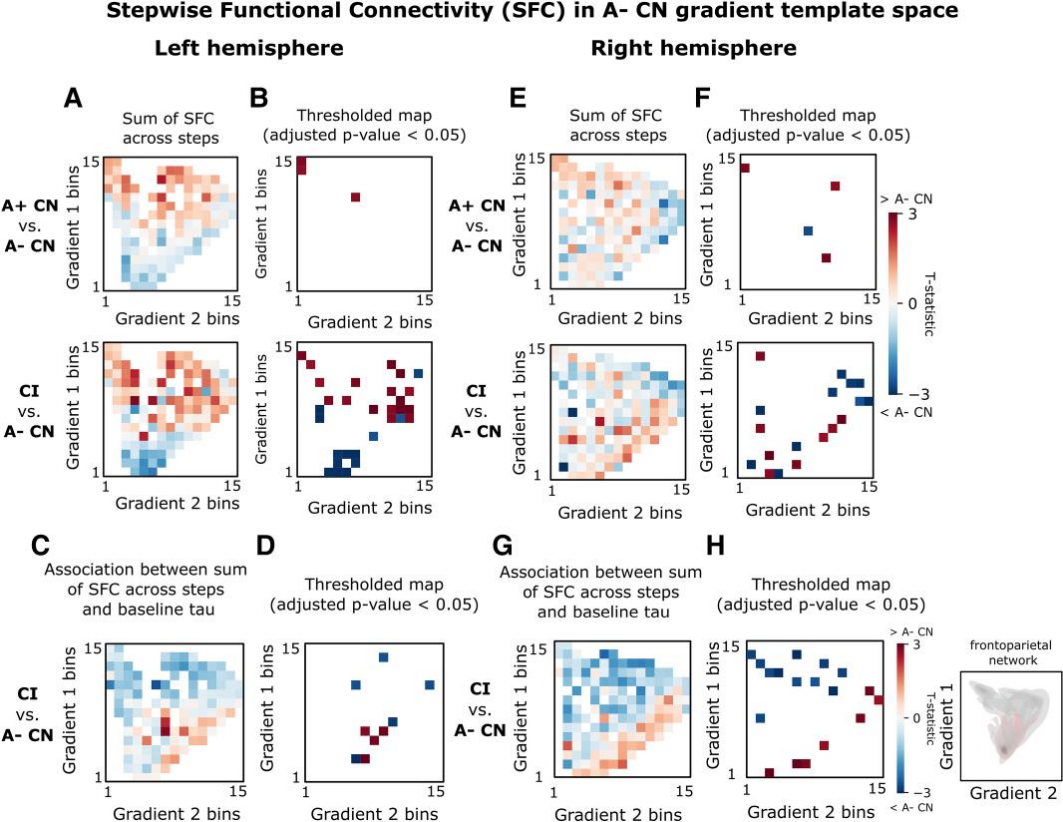
**Correlation between stepwise functional connectivity to EC and tau visualized in gradient space.** (**A**) Groupwise comparisons via a linear regression between SFC summed across all steps for each ROI prior to binning for the left hemisphere (A− CN: *N* = 102, A+ CN: *N* = 35, CI: *N* = 75). (**B**) Thresholded *t*-statistic maps from (**A**) for bins containing ROIs surviving family-wise error correction (adjusted *P*-value < 0.05). (**C**) T-statistic of the linear regression between each ROI’s baseline tau and the ROI-wise between-group differences between the sum of SFC summed across all steps for CI (*N* = 75) and the A− CN (*N* = 102) group average for the left hemisphere. See Supplementary Fig. 11A (left) for this analysis replicated using only steps 1–4 (Fig. 1B). (**D**) Thresholded *t*-statistic maps from (**C**) for bins containing ROIs surviving family-wise error correction (adjusted *P*-value < 0.05). (**E**) Groupwise comparisons via a linear regression between SFC summed across all steps for each ROI prior to binning for the right hemisphere (A− CN: *N* = 102, A+ CN: *N* = 35, CI: *N* = 75). (**F**) Thresholded *t*-statistic maps from (**E**) for bins containing ROIs surviving family-wise error correction (adjusted *P*-value < 0.05). (**G**) T-statistic of the linear regression between each ROI’s baseline tau and the ROI-wise between-group difference between the sum of SFC summed across all steps for CI (*N* = 75) and the A− CN (*N* = 102) group average for the right hemisphere. See Supplementary Fig. 11A (right) for this analysis repeated using only steps 1–4, where the majority of the convergence was observed (Fig. 1B). (**H**) Thresholded *t*-statistic maps from (**G**) for bins containing ROIs surviving family-wise error correction, where the adjusted *P*-value < 0.05. The networks with notable correlation with tau are highlighted in red (right). Color scale indicates t-statistic. Red pixels indicate increased SFC in A+ CN/CI compared with A− CN, while blue pixels indicate reduced SFC in A+ CN/CI compared with A− CN. CN, cognitively normal; CI, cognitively impaired; EC, entorhinal cortex; A− CN, healthy control; ROI, region-of-interest; SFC, stepwise functional connectivity.

**Figure 5:**
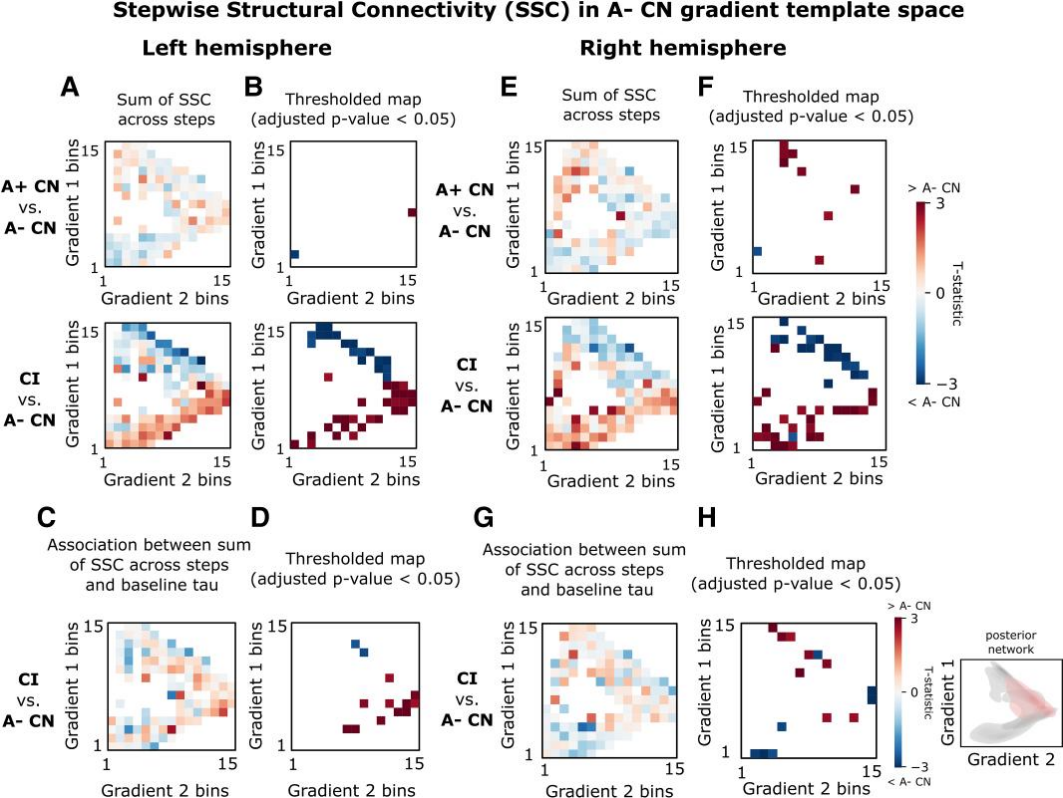
**Correlation between stepwise structural connectivity to EC and tau visualized in gradient space.** (**A**) Groupwise comparisons via a linear regression between SSC summed across all steps for each ROI prior to binning for the left hemisphere (A− CN: *N* = 102, A+ CN: *N* = 35, CI: *N* = 75). (**B**) Thresholded *t*-statistic maps from (**A**) for bins containing ROIs surviving family-wise error correction (adjusted *P*-value < 0.05). (**C**) T-statistic of the linear regression between each ROI’s baseline tau and the ROI-wise between-group difference between the sum of SSC summed across all steps for CI (*N* = 75) and the A− CN (*N* = 102) group average for the left hemisphere. See Supplementary Fig. 11B (left) for this analysis repeated using only steps 1–4 where the majority of the convergence was observed (Fig. 1B). (**D**) Thresholded *t*-statistic maps from (**C**) for bins containing ROIs surviving family-wise error correction (adjusted *P*-value < 0.05). (**E**) Groupwise comparisons via a linear regression between SSC summed across all steps for each ROI prior to binning for the right hemisphere (A− CN: *N* = 102, A+ CN: *N* = 35, CI: *N* = 75). (**F**) Thresholded *t*-statistic maps from (**E**) for bins containing ROIs surviving family-wise error correction (adjusted *P*-value < 0.05). (**G**) T-statistic of the linear regression between each ROI’s baseline tau and the ROI-wise between-group difference between the sum of SSC summed across all steps for CI (*N* = 75) and the A− CN (*N* = 102) group average for the right hemisphere. See Supplementary Fig. 11B (right) for this analysis repeated using only steps 1–4, where the majority of the convergence was observed (Fig. 1B). (**H**) Thresholded *t*-statistic maps from (**G**) for bins containing ROIs surviving family-wise error correction (adjusted *P*-value < 0.05). The networks with notable correlation with tau are highlighted in red (right). Color scale indicates *t*-statistic. Red pixels indicate increased SSC in A+ CN/CI compared with A− CN, while blue pixels indicate reduced SSC in A+ CN/CI compared with A− CN. CN, cognitively normal; CI, cognitively impaired; EC, entorhinal cortex; A− CN, healthy control; ROI, region-of-interest; SSC, stepwise structural connectivity.

Lastly, we investigated the association between baseline tau SUVR and the difference in within-subject normalized SFC/SSC between diagnostic groups. To this end, we summed the normalized SFC across steps and created a heatmap of their groupwise comparisons (*t*-statistic) within gradient space (left hemisphere: Fig. 4A and B, right hemisphere: Fig. 4E and F). In CN A+, tau was positively correlated with SFC to the left EC within the transmodal pole of the functional gradient (Supplementary Fig. 9A and B). In CI, this correlation shifted to the frontoparietal network (left hemisphere: Fig. 4C and D, right hemisphere: Fig. 4G and H). SSC was positively correlated with tau in the posterior network for CI (left hemisphere: Fig. 5C and D, right hemisphere: Fig. 5G and H) and medial-lateral temporal network for CN A+ (Supplementary Fig. 9C and D).

## Discussion

Using an integrated stepwise connectivity and connectome gradient approach of neuroimaging data,^14^ we demonstrated widespread network reorganization in AD, affecting both direct and indirect, multi-step functional and structural connections to the EC. Functional hypoconnectivity to the transmodal pole of the primary functional gradient and structural hypoconnectivity to the posterior pole of the primary structural gradient were observed at direct (one step size) from the EC, while additional inter-network hyperconnectivity emerged at greater step sizes. These connectivity changes were observed relative to the average within-subject brain-wide connectivity. Furthermore, we observed stage-dependent shifts in functional connectivity–tau correlations with disease progression, being stronger in the highest-order transmodal network of the primary functional gradient (DMN/limbic) in the preclinical stage, which then shifted to the second highest-order transmodal network (frontoparietal) in the clinical stage.

Regarding brain-wide functional connectivity of the EC in AD, we demonstrated hypoconnectivity within the DMN (transmodal) but increased connectivity to subnetworks of the frontoparietal and cingulo-opercular (transmodal) and somatomotor (unimodal) cortices. Previous studies of direct ROI-to-ROI connectivity measures demonstrated greater inter-network connectivity or loss of inter-network anti-correlation between the DMN and executive control networks, as well as a loss of intra-network connectivity within the DMN, with greater AD severity.^25-27^ Hyperconnectivity within the sensorimotor network and to the DMN has also been demonstrated in older healthy adults compared with young healthy adults using the SFC approach.^28^ Through a novel connectome gradient approach, we showed increases in stepwise connectivity (relative to the whole brain) between the EC (transmodal) and both transmodal and unimodal regions that become more prominent for indirect (imaging-derived) connections. On the other hand, for structural connectivity of the EC, we demonstrated decreases in stepwise connectivity within the posterior cortex but increased stepwise connectivity to the somatomotor and anterior cortices. This suggests that the temporal-posterior structural degeneration observed in AD may be accompanied by an increase in the importance of intact long-range connections to the EC, particularly those involving medial-anterior cortices. Taken together, our work unveils how AD affects connectivity strength along the transmodal-unimodal (functional) and posterior-anterior (structural) axes of brain organization.

Our findings in A+ CN were suggestive of predominantly hyperconnectivity of the EC to the neocortex within the functional gradient space and the posterior end of the structural gradient space, which may be compensatory hyperconnectivity in preclinical stages.^29-31^ Compensatory connectivity patterns have also been observed in Parkinson’s disease (PD). Increased coupling amongst the DMN, fronto-parietal, visual, and salience networks was associated with better initial cognitive outcomes in PD patients who were CN.^32^ However, the increased coupling also predicted a greater decline in cognition.^32^ Alternatively, the hyperconnectivity observed could be a pathological aberrant synchronization between the DMN and the rest of the brain, as suggested by Malotaux *et al*.^33^ Our findings align with the hypothesis that internetwork hyperconnectivity could be a hallmark of both preclinical and late disease stages, where the network reorganization may help preserve cognitive function temporarily.^29,34^ Alternatively, increases in functional connectivity may also be interpreted as an abnormal synchronization network de-differentiation, in line with our recent work^4^ rather than a compensation mechanism.^33^ However, we also acknowledge that the connectivity increases might be due to the underlying decrease in structural connectivity in AD-signature regions, which could cause functional connectivity to appear increased, both within networks and globally.

Interestingly, our results suggest more pronounced lateralization in functional compared with structural network reorganization in AD. In the left hemisphere, there was increased and decreased connectivity of the EC with unimodal and transmodal areas (relative to the whole brain), respectively, while a divergent trend was observed for the right hemisphere. We speculate that these left-right differences may be related to the greater intra-network connectivity in language regions in the left hemisphere and/or greater left medial-temporal lobe (MTL) atrophy.^35^ In contrast to our observed lateralization in connectivity, the association between SFC and tau showed similar patterns for both hemispheres. This is in line with a recent study by Anijärv *et al*.,^36^ showing that asymmetry in tau was related to asymmetry in amyloid rather than connectivity.

We observed a correlation of SFC with tau-PET in the highest-order cognitive network of the transmodal cortex (i.e. DMN) in the preclinical stage, which shifted to the second-highest order cognitive network of the transmodal cortex (i.e. fronto-parietal) in the clinical stage. Similarly, we observed a correlation of SSC with tau-PET in the posterior end (MTL) of the structural gradient space in the preclinical stage, which shifted more temporo-occipital in the clinical stage. These ‘stage-dependent shifts’ in tau–connectivity correlations within gradient space as cognitive decline advances may reflect a tau-mediated weakening of the DMN (functional) and MTL (structural) connectivity, together with an engagement of alternative long-range hub connections. This notion is in line with a graph theory study in AD,^37^ which found that increased global tau burden in the clinical stage was associated with a greater functional influence of frontal regions on the rest of the cortex, characterized by more direct long-range connections through fewer nodes, and at the expense of reduced local efficiency.

Our study has some limitations. We note that SFC/SSC was calculated at the subject level but represented in template gradient space generated from the mean of all subjects in the group. This means that the variation in the subjects’ gradient space was not fully captured in this analysis, which could have diminished the effect sizes of the group-wise comparisons. Finally, while the three diagnostic groups may represent different stages of disease progression, the data are ultimately cross-sectional. Future work can consider the relationship between stepwise connectivity, gradients, and various neurophysiological aspects of cognition to better translate findings into clinical implications. Additionally, the study can be replicated in more diverse, longitudinal cohorts.

In summary, we demonstrated network reorganization in AD affecting both direct and indirect, multi-step functional and structural connections between the EC and the rest of the brain. Integrating stepwise connectivity and connectome gradient space allows a better understanding of how AD affects connectivity along the major axes of brain organization.

## Supplementary Material

fcaf399_Supplementary_Data

## Data Availability

All requests for raw and analyzed data and materials will be promptly reviewed by McGill University to verify if the request is subject to any intellectual property or confidentiality obligations. Anonymized data will be shared upon request to the study’s senior author from a qualified academic investigator for the sole purpose of replicating the procedures and results presented in this article. Any data and materials that can be shared will be released via a material transfer agreement. Data are not publicly available due to information that could compromise the privacy of research participants. Related documents, including study protocol and informed consent forms, can similarly be made available upon request. Code for stepwise connectivity analyses and reproduction of figures can be found at https://github.com/AICONSlab/stepwise_connectivity.git.

## References

[1] Vogel JW, Iturria-Medina Y, Strandberg OT, et al Spread of pathological tau proteins through communicating neurons in human Alzheimer’s disease. Nat Commun. 2020;11(1):2612. 32457389 10.1038/s41467-020-15701-2 PMC7251068

[2] Zhou J, Gennatas ED, Kramer JH, Miller BL, Seeley WW. Predicting regional neurodegeneration from the healthy brain functional connectome. Neuron. 2012;73(6):1216–1227. 22445348 10.1016/j.neuron.2012.03.004 PMC3361461

[3] Guo JL, Narasimhan S, Changolkar L, et al Unique pathological tau conformers from Alzheimer’s brains transmit tau pathology in nontransgenic mice. J Exp Med. 2016;213(12):2635–2654. 27810929 10.1084/jem.20160833 PMC5110027

[4] Ottoy J, Kang MS, Tan JXM, et al Tau follows principal axes of functional and structural brain organization in Alzheimer’s disease. Nat Commun. 2024;15(1):5031. 38866759 10.1038/s41467-024-49300-2 PMC11169286

[5] Therriault J, Pascoal TA, Lussier FZ, et al Biomarker modeling of Alzheimer’s disease using PET-based Braak staging. Nat Aging. 2022;2(6):526–535. 37118445 10.1038/s43587-022-00204-0 PMC10154209

[6] Braak H, Braak E. Neuropathological stageing of Alzheimer-related changes. Acta Neuropathol. 1991;82(4):239–259. 1759558 10.1007/BF00308809

[7] Sanz-Arigita EJ, Schoonheim MM, Damoiseaux JS, et al Loss of ‘small-world’ networks in Alzheimer’s disease: Graph analysis of FMRI resting-state functional connectivity. PLoS One. 2010;5(11):e13788. 21072180 10.1371/journal.pone.0013788 PMC2967467

[8] Sepulcre J, Grothe MJ, d’Oleire Uquillas F, et al Neurogenetic contributions to amyloid beta and tau spreading in the human cortex. Nat Med. 2018;24(12):1910–1918. 30374196 10.1038/s41591-018-0206-4 PMC6518398

[9] Basaia S, Agosta F, Francia A, et al Cerebro-cerebellar motor networks in clinical subtypes of Parkinson’s disease. NPJ Parkinsons Dis. 2022;8(1):113. 36068246 10.1038/s41531-022-00377-w PMC9448730

[10] Sepulcre J, Sabuncu MR, Becker A, Sperling R, Johnson KA. In vivo characterization of the early states of the amyloid-beta network. Brain. 2013;136(Pt 7):2239–2252. 23801740 10.1093/brain/awt146 PMC3692037

[11] Costumero V, d’Oleire Uquillas F, Diez I, et al Distance disintegration delineates the brain connectivity failure of Alzheimer’s disease. Neurobiol Aging. 2020;88:51–60. 31941578 10.1016/j.neurobiolaging.2019.12.005 PMC7085436

[12] Margulies DS, Ghosh SS, Goulas A, et al Situating the default-mode network along a principal gradient of macroscale cortical organization. Proc Natl Acad Sci U S A. 2016;113(44):12574–12579. 27791099 10.1073/pnas.1608282113 PMC5098630

[13] Smallwood J, Bernhardt BC, Leech R, Bzdok D, Jefferies E, Margulies DS. The default mode network in cognition: A topographical perspective. Nat Rev Neurosci. 2021;22(8):503–513. 34226715 10.1038/s41583-021-00474-4

[14] Paquola C, Amunts K, Evans A, Smallwood J, Bernhardt B. Closing the mechanistic gap: The value of microarchitecture in understanding cognitive networks. Trends Cogn Sci. 2022;26(10):873–886. 35909021 10.1016/j.tics.2022.07.001

[15] Dong D, Yao D, Wang Y, et al Compressed sensorimotor-to-transmodal hierarchical organization in schizophrenia. Psychol Med. 2023;53(3):771–784. 34100349 10.1017/S0033291721002129

[16] Hong SJ, Vos de Wael R, Bethlehem RAI, et al Atypical functional connectome hierarchy in autism. Nat Commun. 2019;10(1):1022. 30833582 10.1038/s41467-019-08944-1 PMC6399265

[17] Pascoal TA, Benedet AL, Ashton NJ, et al Microglial activation and tau propagate jointly across Braak stages. Nat Med. 2021;27(9):1592–1599. 34446931 10.1038/s41591-021-01456-w

[18] Esteban O, Markiewicz CJ, Blair RW, et al fMRIPrep: A robust preprocessing pipeline for functional MRI. Nat Methods. 2019;16(1):111–116. 30532080 10.1038/s41592-018-0235-4 PMC6319393

[19] Tournier JD, Smith R, Raffelt D, et al MRtrix3: A fast, flexible and open software framework for medical image processing and visualisation. Neuroimage. 2019;202:116137. 31473352 10.1016/j.neuroimage.2019.116137

[20] Glasser MF, Coalson TS, Robinson EC, et al A multi-modal parcellation of human cerebral cortex. Nature. 2016;536(7615):171–178. 27437579 10.1038/nature18933 PMC4990127

[21] Vos de Wael R, Benkarim O, Paquola C, et al BrainSpace: A toolbox for the analysis of macroscale gradients in neuroimaging and connectomics datasets. Commun Biol. 2020;3(1):103. 32139786 10.1038/s42003-020-0794-7 PMC7058611

[22] Sepulcre J, Sabuncu MR, Yeo TB, Liu H, Johnson KA. Stepwise connectivity of the modal cortex reveals the multimodal organization of the human brain. J Neurosci. 2012;32(31):10649–10661. 22855814 10.1523/JNEUROSCI.0759-12.2012 PMC3483645

[23] Pretus C, Marcos-Vidal L, Martínez-García M, et al Stepwise functional connectivity reveals altered sensory-multimodal integration in medication-naïve adults with attention deficit hyperactivity disorder. Hum Brain Mapp. 2019;40(16):4645–4656. 31322305 10.1002/hbm.24727 PMC6865796

[24] Vogel JW, Young AL, Oxtoby NP, et al Four distinct trajectories of tau deposition identified in Alzheimer’s disease. Nat Med. 2021;27(5):871–881. 33927414 10.1038/s41591-021-01309-6 PMC8686688

[25] Lin Q, Rosenberg MD, Yoo K, Hsu TW, O’Connell TP, Chun MM. Resting-state functional connectivity predicts cognitive impairment related to Alzheimer’s disease. Front Aging Neurosci. 2018;10:94. 29706883 10.3389/fnagi.2018.00094 PMC5908906

[26] Prado P, Moguilner S, Mejía JA, et al Source space connectomics of neurodegeneration: One-metric approach does not fit all. Neurobiol Dis. 2023;179:106047. 36841423 10.1016/j.nbd.2023.106047 PMC11170467

[27] Penalba-Sánchez L, Oliveira-Silva P, Sumich AL, Cifre I. Increased functional connectivity patterns in mild Alzheimer’s disease: A rsfMRI study. Front Aging Neurosci. 2022;14:1037347. 36698861 10.3389/fnagi.2022.1037347 PMC9869068

[28] Filippi M, Cividini C, Basaia S, et al Age-related vulnerability of the human brain connectome. Mol Psychiatry. 2023;28(12):5350–5358. 37414925 10.1038/s41380-023-02157-1 PMC11041755

[29] Schultz AP, Chhatwal JP, Hedden T, et al Phases of hyperconnectivity and hypoconnectivity in the default mode and salience networks track with amyloid and Tau in clinically normal individuals. J Neurosci. 2017;37(16):4323–4331. 28314821 10.1523/JNEUROSCI.3263-16.2017 PMC5413178

[30] Jack CR Jr, Wiste HJ, Weigand SD, et al Amyloid-first and neurodegeneration-first profiles characterize incident amyloid PET positivity. Neurology. 2013;81(20):1732–1740. 24132377 10.1212/01.wnl.0000435556.21319.e4 PMC3821718

[31] Dickerson BC, Salat DH, Greve DN, et al Increased hippocampal activation in mild cognitive impairment compared to normal aging and AD. Neurology. 2005;65(3):404–411. 16087905 10.1212/01.wnl.0000171450.97464.49 PMC4335677

[32] Wei X, Shen Q, Litvan I, Huang M, Lee RR, Harrington DL. Internetwork connectivity predicts cognitive decline in Parkinson’s and is altered by genetic variants. Front Aging Neurosci. 2022;14:853029. 35418853 10.3389/fnagi.2022.853029 PMC8996114

[33] Malotaux V, Dricot L, Quenon L, Lhommel R, Ivanoiu A, Hanseeuw B. Default-mode network connectivity changes during the progression toward Alzheimer’s dementia: A longitudinal functional magnetic resonance imaging study. Brain Connect. 2023;13(5):287–296. 36103377 10.1089/brain.2022.0008

[34] Vervoort G, Alaerts K, Bengevoord A, et al Functional connectivity alterations in the motor and fronto-parietal network relate to behavioral heterogeneity in Parkinson’s disease. Parkinsonism Relat Disord. 2016;24:48–55. 26924603 10.1016/j.parkreldis.2016.01.016

[35] Lubben N, Ensink E, Coetzee GA, Labrie V. The enigma and implications of brain hemispheric asymmetry in neurodegenerative diseases. Brain Commun. 2021;3(3):fcab211. 10.1093/braincomms/fcab211 PMC8454206 34557668

[36] Anijärv TE, Ossenkoppele R, Smith R, et al Hemispheric asymmetry of tau pathology is related to asymmetric amyloid deposition in Alzheimer’s disease. Nat Commun. 2025;16:8232. 40913038 10.1038/s41467-025-63564-2 PMC12413461

[37] Cope TE, Rittman T, Borchert RJ, et al Tau burden and the functional connectome in Alzheimer’s disease and progressive supranuclear palsy. Brain. 2018;141(2):550–567. 29293892 10.1093/brain/awx347 PMC5837359

