# Supplementary material for: Stepwise connectivity of the entorhinal cortex along connectomic gradients in Alzheimer’s disease
Source: Brain Commun. 2025 Oct 14;7(6):fcaf399. doi: 10.1093/braincomms/fcaf399 (PMC12585350; doi:10.1093/braincomms/fcaf399)
Supplement: fcaf399_Supplementary_Data [file fcaf399_supplementary_data.docx]

### Supplementary Figures

**
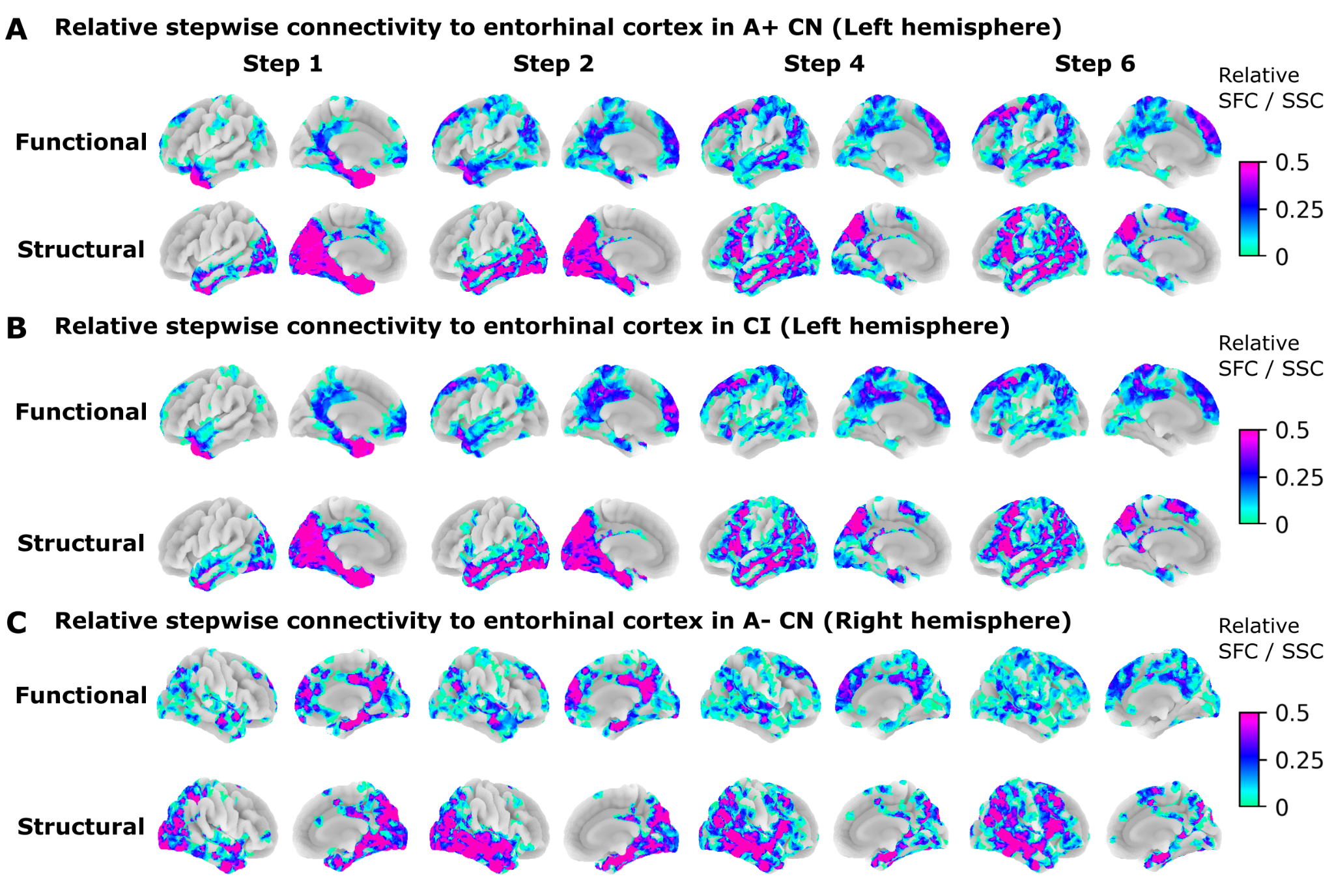
**

**Supplementary Figure 1: Whole-brain stepwise connectivity to the entorhinal cortex seed.** **(A) and (B)** Group averaged normalized SFC and SSC to the left EC for A+ CN (N=35) **(A)** and CI (N=75) **(B)**, see Fig. 1 for A- CN. Group averaged normalized SFC and SSC to the right EC for A- CN (N=103) **(C)**. The SFC/SCC maps were within-subject normalized across ROIs to indicate stepwise changes relative to the rest of the brain. CI: cognitively impaired; CN: cognitively normal; EC: entorhinal cortex; A- CN: healthy control; SFC: stepwise functional connectivity; SSC: stepwise structural connectivity.

**
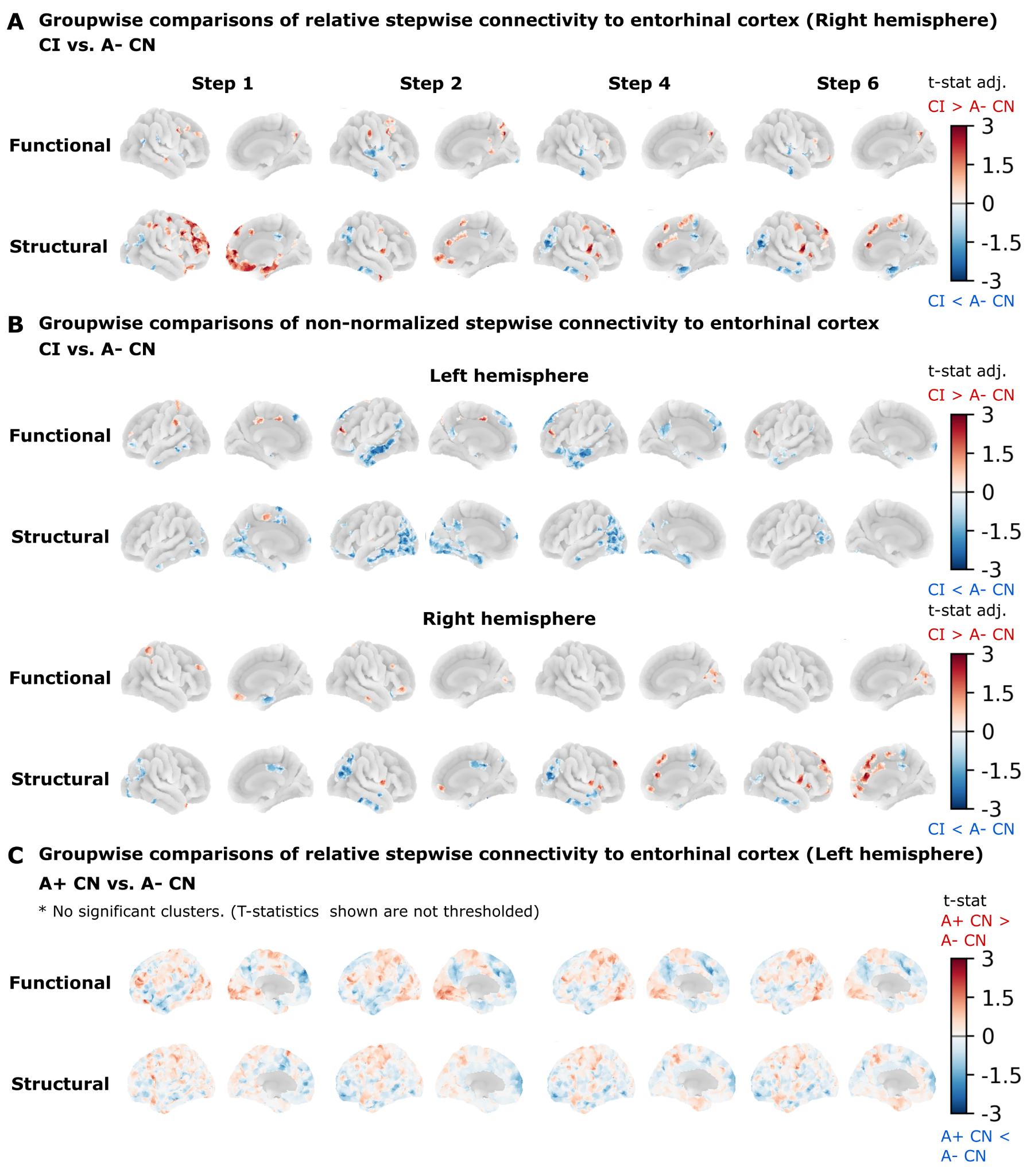
**

**Supplementary Figure 2: ROI-wise groupwise comparisons of whole-brain stepwise connectivity to the entorhinal cortex seed.**  **(A)** Groupwise comparisons via a linear regression of within-subject normalized to whole brain SFC and SSC in CI (N=75) compared to A- CN (N=103). Target ROIs with increased SFC/SSC in CI compared to A- CN are shown in red, while target ROIs with reduced SFC/SSC in CI compared to A- CN are shown in blue. Only target ROIs with significant p-values after adjustment are shown. See Fig. 1 for left hemisphere results. **(B)** Groupwise comparisons via a linear regression of non-within-subject normalized stepwise connectivity to the EC for the left (top) and right (bottom) hemispheres. **(C)** Groupwise comparisons via a linear regression of within-subject normalized SFC and SSC in A+ CN (N=35) compared to A- CN (N=103). As no significant clusters survived correction for family-wise errors, unthresholded t-statistic maps are shown. CI: cognitively impaired; CN: cognitively normal; EC: entorhinal cortex; SFC: stepwise functional connectivity; SSC: stepwise structural connectivity.


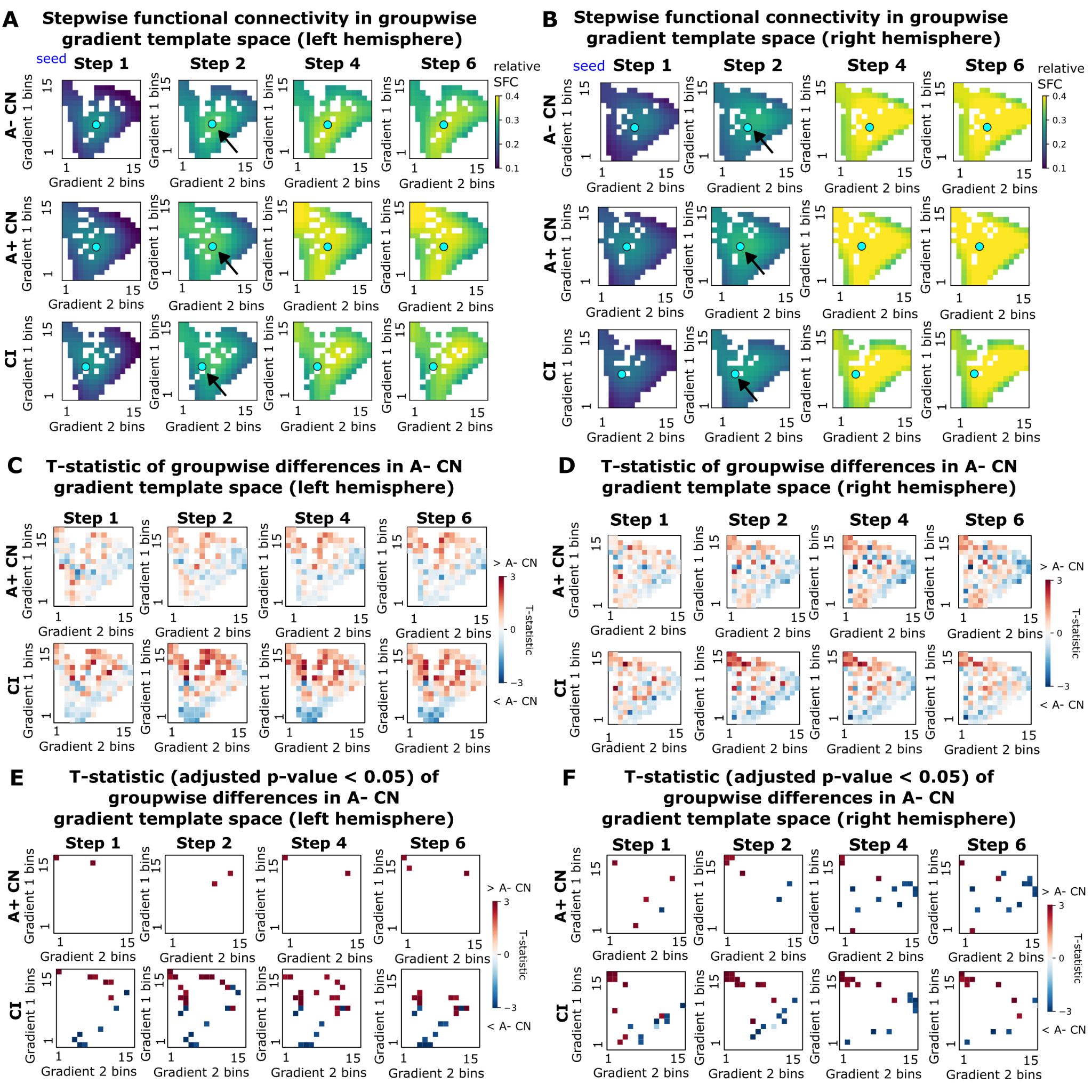


**Supplementary Figure 3: Stepwise functional connectivity of the posterior inferotemporal region visualized along the axes of the principal gradients of the brain.** **(A) and (B)** Within-subject normalized (across ROIs) SFC to the posterior inferotemporal region projected in the template functional gradient space for each group (A- CN: N=103, A+ CN: N=35, CI:N=75), for the left **(A)** and right **(B)** hemispheres. Both gradients have been min-max scaled to range from 0 to 1 to allow direct comparison between groups. Cyan marker and black arrow indicate the location of the posterior inferotemporal region seed. **(C) and (D)** ROI-wise group comparisons via a linear regression of SFC for A+ CN (N=35) / CI (N=75) with A- CN (N=103) group. Color scale indicates t-statistic. Red pixels indicate increased SFC in A+ CN/CI compared to A- CN, while blue pixels indicate reduced SFC in A+ CN/CI compared to A- CN. **(E) and (F)** T-statistics surviving correction for family-wise errors where the adjusted p-value < 0.05. CN: cognitively normal; CI: cognitively impaired; ROI: region-of-interest; SFC: stepwise functional connectivity.


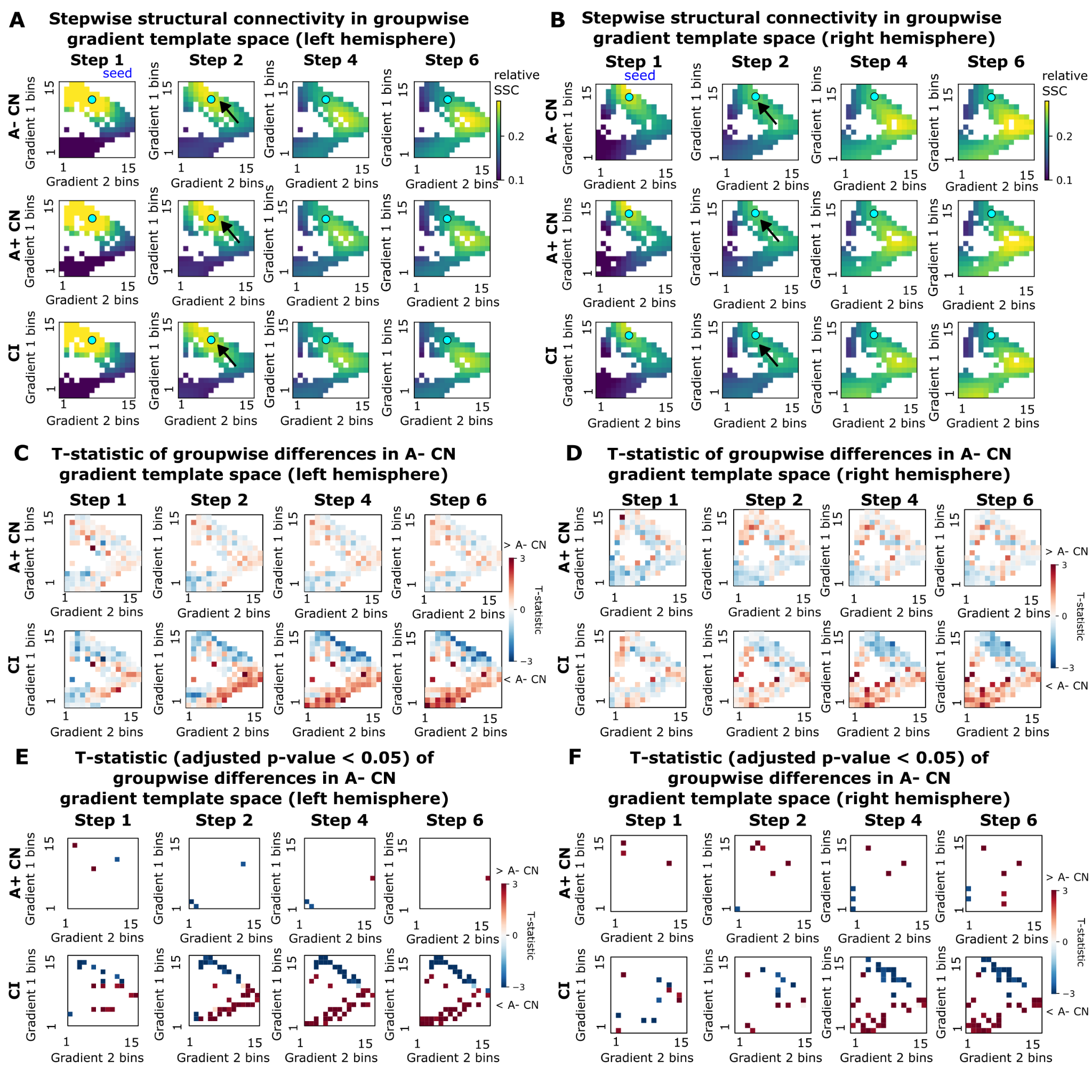


**Supplementary Figure 4: Stepwise structural connectivity of the posterior inferotemporal region visualized along the axes of the principal gradients of the brain.** **(A) and (B)** Within-subject normalized (across ROIs) SFC to the posterior inferotemporal region projected in the template structural gradient space for each group (A- CN: N=103, A+ CN: N=35, CI:N=75), for the left **(A)** and right **(B)** hemispheres. Both gradients have been min-max scaled to range from 0 to 1 to allow direct comparison between groups. Cyan marker and black arrow indicate the location of the posterior inferotemporal region seed. **(C) and (D)** ROI-wise group comparisons via a linear regression of SSC for A+ CN (N=35) / CI (N=75) with A- CN (N=103) group. Color scale indicates t-statistic. Red pixels indicate increased SSC in A+ CN/CI compared to A- CN, while blue pixels indicate reduced SFC in A+ CN/CI compared to A- CN. **(E) and (F)** T-statistics surviving correction for family-wise errors where the adjusted p-value < 0.05. CN: cognitively normal; CI: cognitively impaired; ROI: region-of-interest; SSC: stepwise structural connectivity.


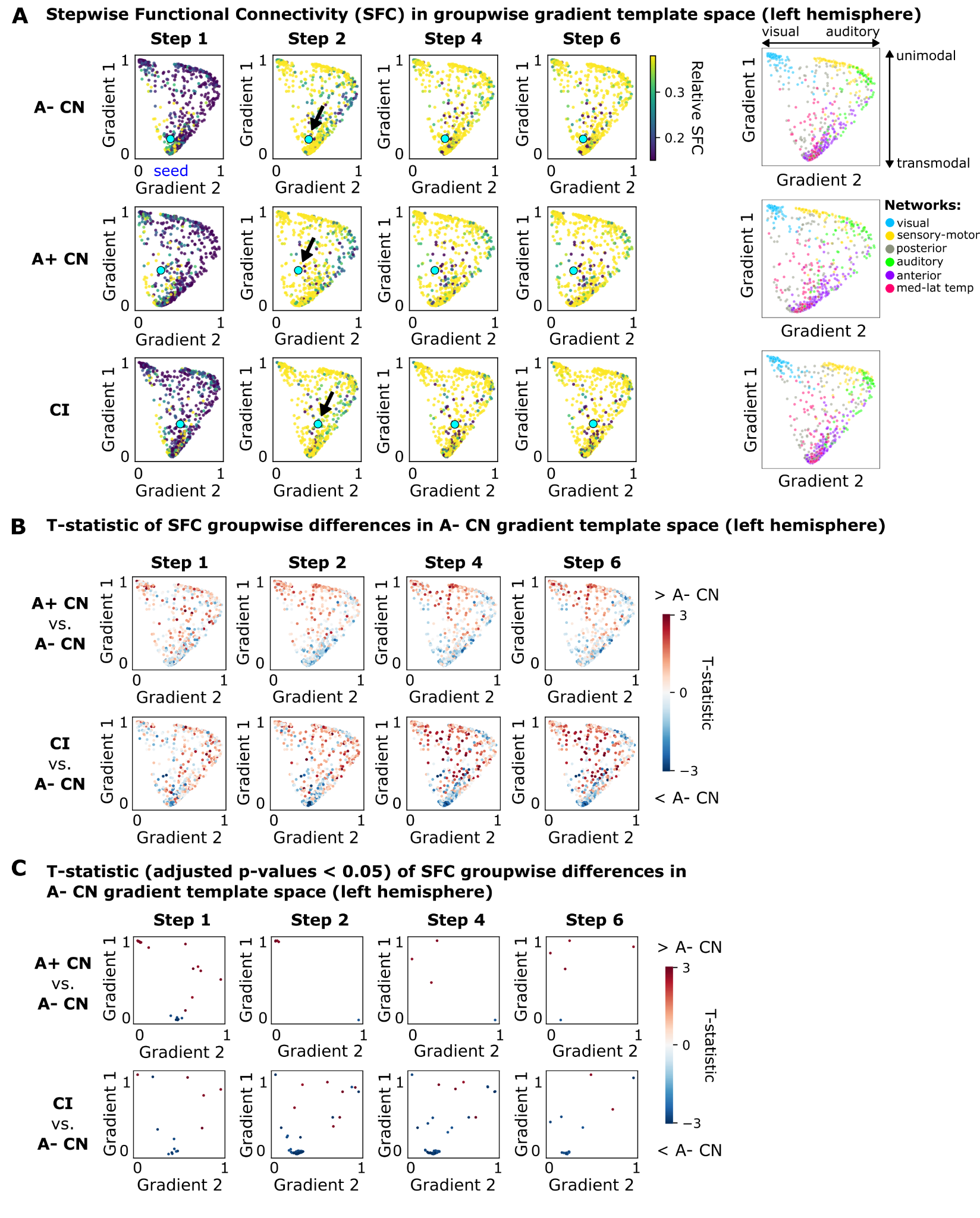


**Supplementary Figure 5: Stepwise functional connectivity of left EC to left hemisphere visualized along the axes of the principal gradients of the brain, without smoothing of heatmaps.** Within-subject normalized SFC to the EC projected in the template functional **(A)** gradient space for each group (A- CN: N=103, A+ CN: N=35, CI:N=75). Scatterplot of target ROIs are colored by major networks projected in the template functional gradient space of each group (right). Cyan marker and black arrow indicate the location of the EC seed. See Fig. 2 for the smoothed version. **(B)** Groupwise comparisons of SFC via a linear regression (A- CN: N=103, A+ CN: N=35, CI: N=75). **(C)** T-statistics surviving correction for family-wise errors where the adjusted p-value < 0.05. Color scale indicates t-statistic. ROIs with increased SFC in A+ CN/CI compared to A- CN are shown in red while ROIs with reduced SFC in A+ CN/CI compared to A- CN are shown in blue. CI: cognitively impaired; CN: cognitively normal; EC: entorhinal cortex; A- CN: healthy control; ROI: region-of-interest; SFC: stepwise functional connectivity.


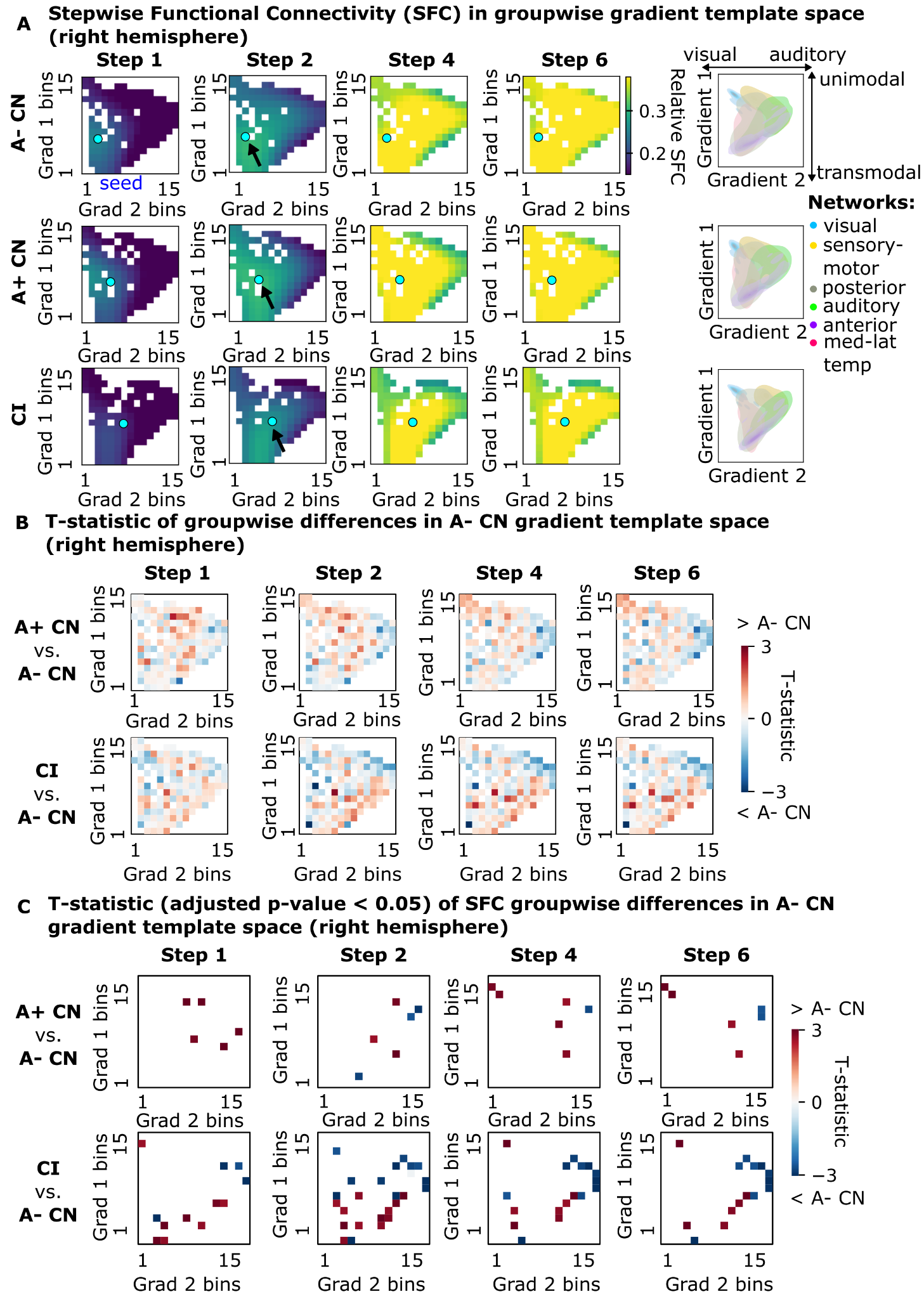


**Supplementary Figure 6: Stepwise functional connectivity of right EC to right hemisphere visualized along the axes of the gradients of the brain.** **(A)** Within-subject normalized SFC to the EC projected in the template functional gradient space for each group (A- CN: N=103, A+ CN: N=35, CI:N=75). ROIs are smoothed into 15 bins along each gradient for visualization. Cyan marker and black arrow indicate the location of the EC seed. Density plots of target ROIs colored by major functional networks projected in the template functional gradient space of each group (right). Both gradients have been min-max scaled to range from 0 to 1 to allow direct comparison between groups. **(B)** Groupwise comparisons of SFC via a linear regression (A- CN: N=103, A+ CN: N=35, CI: N=75). **(C)** T-statistics surviving correction for family-wise errors where the adjusted p-value < 0.05. Color scale indicates t-statistic. Pixels where there were increased SFC in A+ CN/CI compared to A- CN are shown in red while pixels where there were reduced SFC in A+ CN/CI compared to A- CN are shown in blue. CN: cognitively normal; CI: cognitively impaired; EC: entorhinal cortex; ROI: region-of-interest; SFC: stepwise functional connectivity.


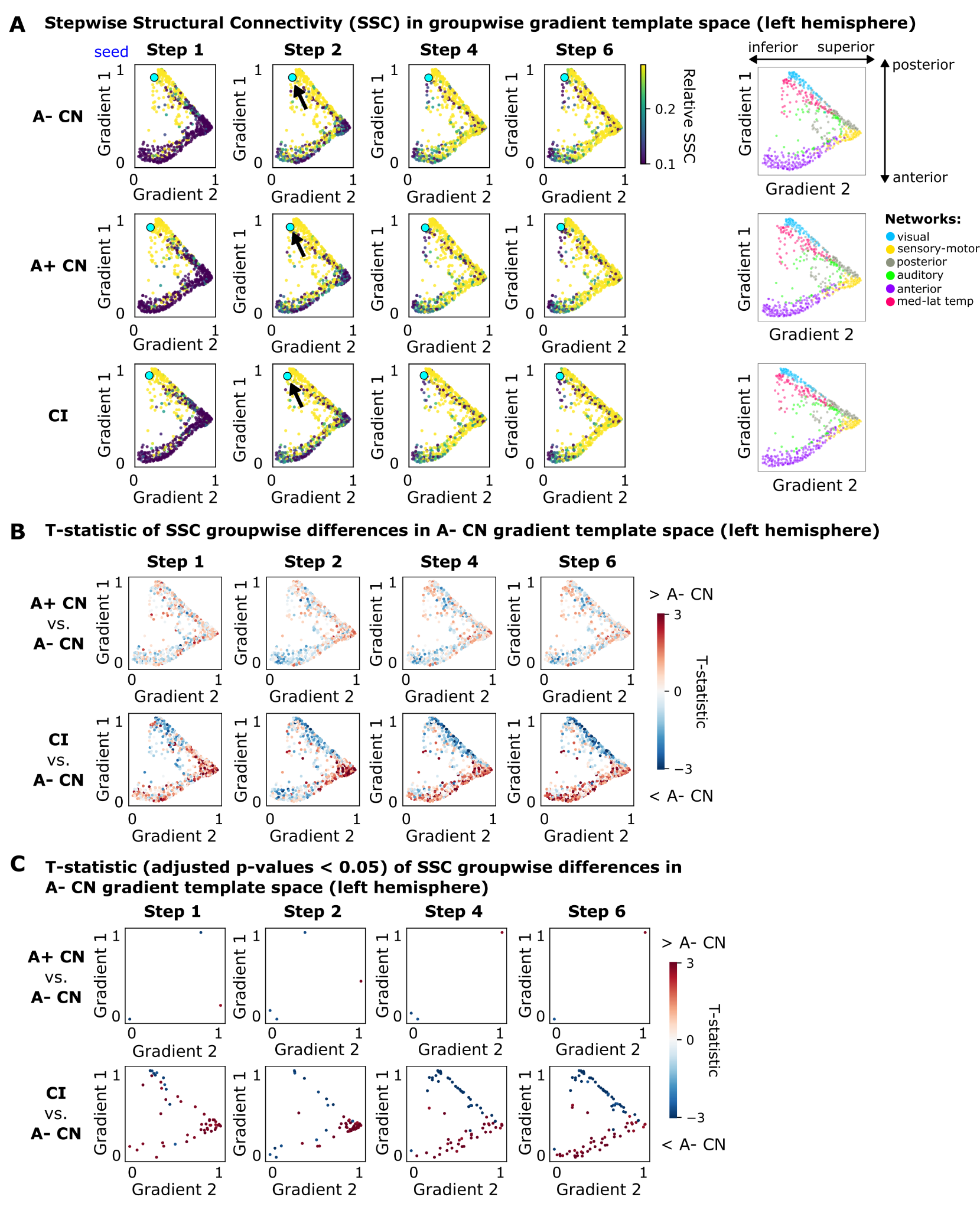


**Supplementary Figure 7: Stepwise structural connectivity of left EC to left hemisphere visualized along the axes of the principal gradients of the brain, without smoothing of heatmaps.** Within-subject normalized SSC to the EC projected in the template structural **(A)** gradient space for each group (A- CN: N=103, A+ CN: N=35, CI:N=75). Scatterplot of target ROIs are colored by major networks projected in the template structural gradient space of each group (right). Cyan marker and black arrow indicate the location of the EC seed. See Fig. 3 for the smoothed version. **(B)** Groupwise comparisons of SSC via a linear regression (A- CN: N=103, A+ CN: N=35, CI: N=75). **(C)** T-statistics surviving correction for family-wise errors where the adjusted p-value < 0.05. Color scale indicates t-statistic. ROIs with increased SSC in A+ CN/CI compared to A- CN are shown in red while ROIs with reduced SSC in A+ CN/CI compared to A- CN are shown in blue. CI: cognitively impaired; CN: cognitively normal; EC: entorhinal cortex; A- CN: healthy control; ROI: region-of-interest; SSC: stepwise structural connectivity.


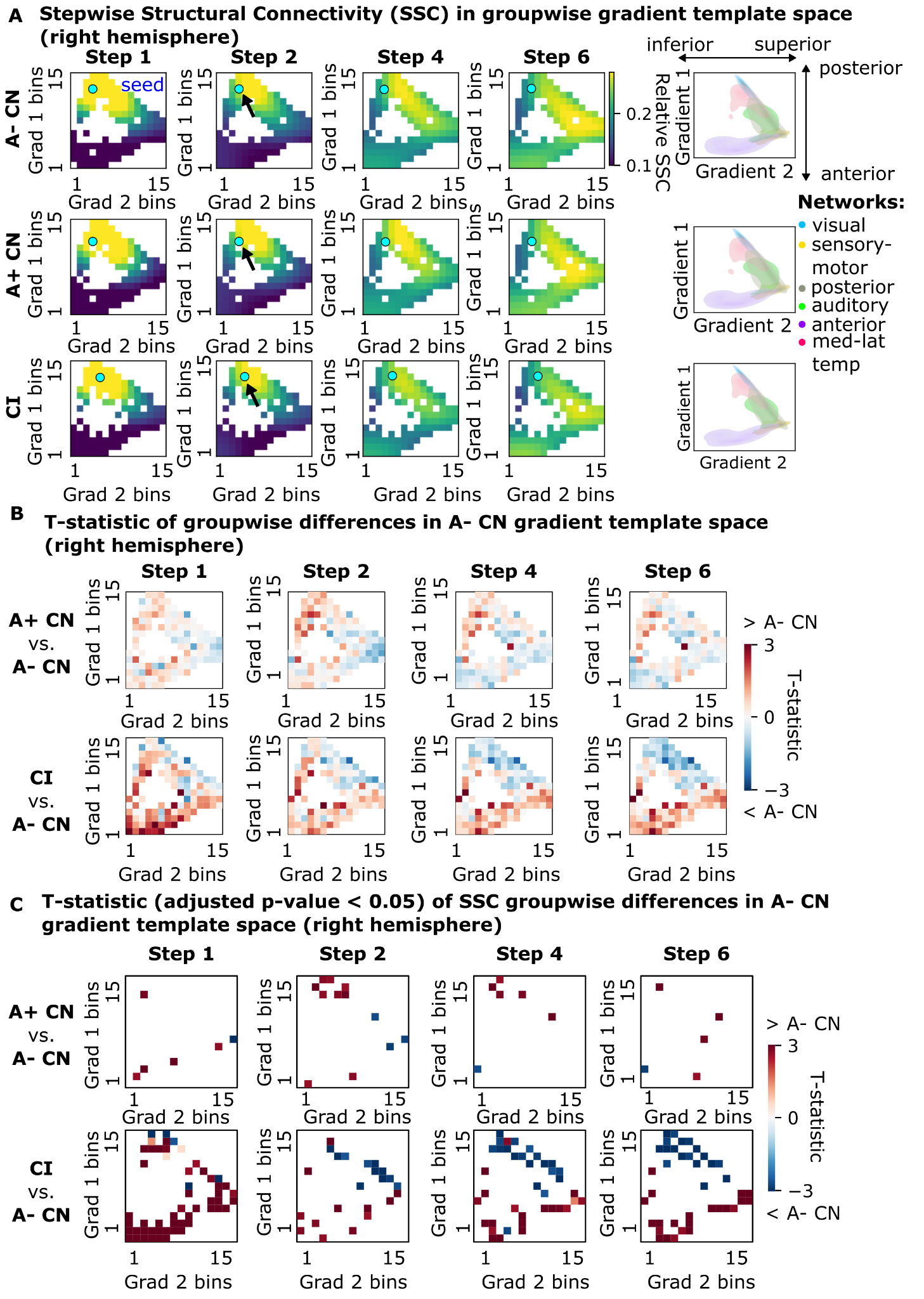


**Supplementary Figure 8: Stepwise structural connectivity of right EC to right hemisphere visualized along the axes of the gradients of the brain.** **(A)** Within-subject normalized SSC to the EC projected in the template functional gradient space for each group (A- CN: N=103, A+ CN: N=35, CI:N=75). ROIs are smoothed into 15 bins along each gradient for visualization. Cyan marker and black arrow indicate the location of the EC seed. Density plots of target ROIs colored by major functional networks projected in the template functional gradient space of each group (right). Both gradients have been min-max scaled to range from 0 to 1 to allow direct comparison between groups. **(B)** Groupwise comparisons of SSC via a linear regression (A- CN: N=103, A+ CN: N=35, CI: N=75). **(C)** T-statistics surviving correction for family-wise errors where the adjusted p-value < 0.05. Color scale indicates t-statistic. Pixels where there were increased SSC in A+ CN/CI compared to A- CN are shown in red while pixels where there were reduced SSC in A+ CN/CI compared to A- CN are shown in blue. CN: cognitively normal; CI: cognitively impaired; EC: entorhinal cortex; ROI: region-of-interest; SSC: stepwise structural connectivity.


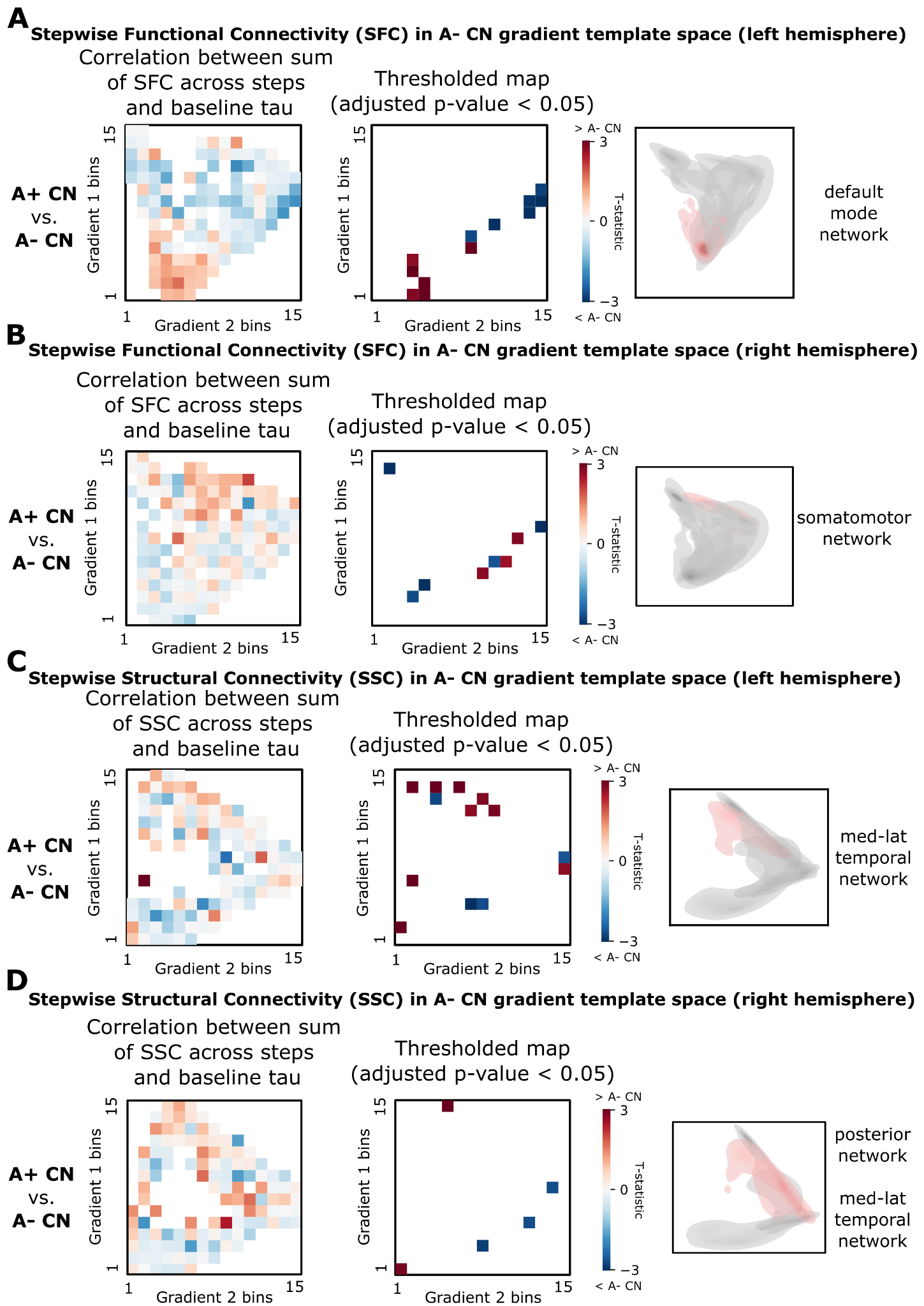


**Supplementary Figure 9: Correlation between stepwise connectivity to EC and tau for the A+ CN group visualized in gradient space. (A)** Groupwise comparisons via a linear regression between SFC summed across all steps for each ROI prior to binning for the left hemisphere (A- CN: N=102, A+ CN: N=35) (left) and thresholded t-statistic maps for bins containing ROIs surviving family-wise error correction where the adjusted p-value < 0.05 (right). **(B)** Groupwise comparisons via a linear regression between SFC summed across all steps for each ROI prior to binning for the right hemisphere (A- CN: N=102, A+ CN: N=35) (left) and thresholded t-statistic maps for bins containing ROIs surviving family-wise error correction where the adjusted p-value < 0.05 (right). **(C)** Groupwise comparisons via a linear regression between SSC summed across all steps for each ROI prior to binning for the left hemisphere (A- CN: N=102, A+ CN: N=35) (left) and thresholded t-statistic maps for bins containing ROIs surviving family-wise error correction where the adjusted p-value < 0.05 (right). **(D)** Groupwise comparisons via a linear regression between SSC summed across all steps for each ROI prior to binning for the right hemisphere (A- CN: N=102, A+ CN: N=35) (left) and thresholded t-statistic maps for bins containing ROIs surviving family-wise error correction where the adjusted p-value < 0.05 (right). The networks with notable correlation with tau are highlighted in red (right). Color scale indicates t-statistic. Red pixels indicate increased SFC/SSC in A+ CN compared to A- CN, while blue pixels indicate reduced SFC/SSC in A+ CN compared to A- CN. CN: cognitively normal; EC: entorhinal cortex; A- CN: healthy control; ROI: region-of-interest; SFC: stepwise functional connectivity; SSC: stepwise structural connectivity.


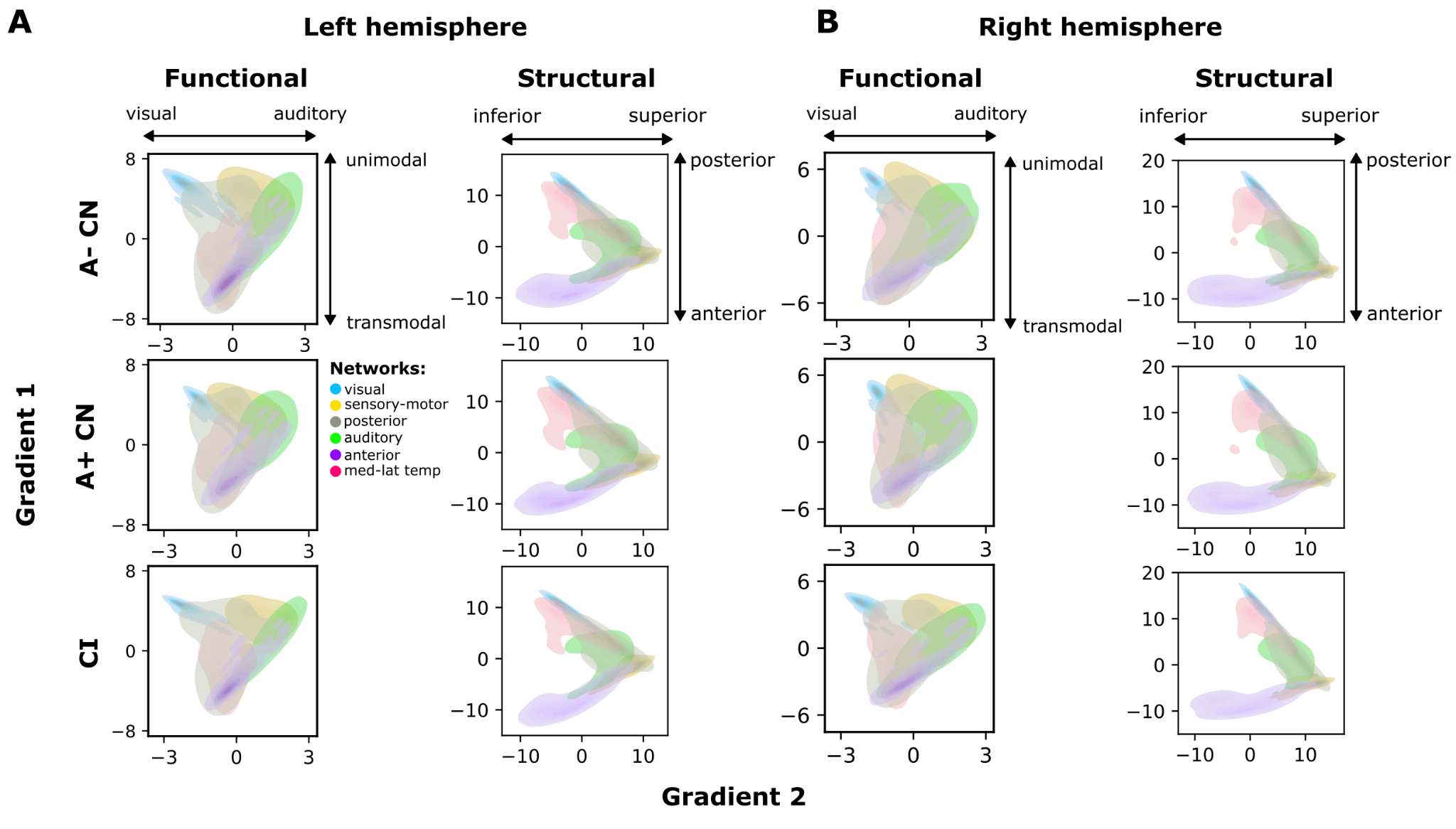


**Supplementary Figure 10:** Density plots of target ROIs colored by major functional networks projected in the template functional and structural gradient space of each group for the left (**A**) and right (**B**) hemispheres. See Fig. 2 and 3 for versions where the gradients have been min-max scaled to range from 0 to 1 to allow direct comparison between groups.


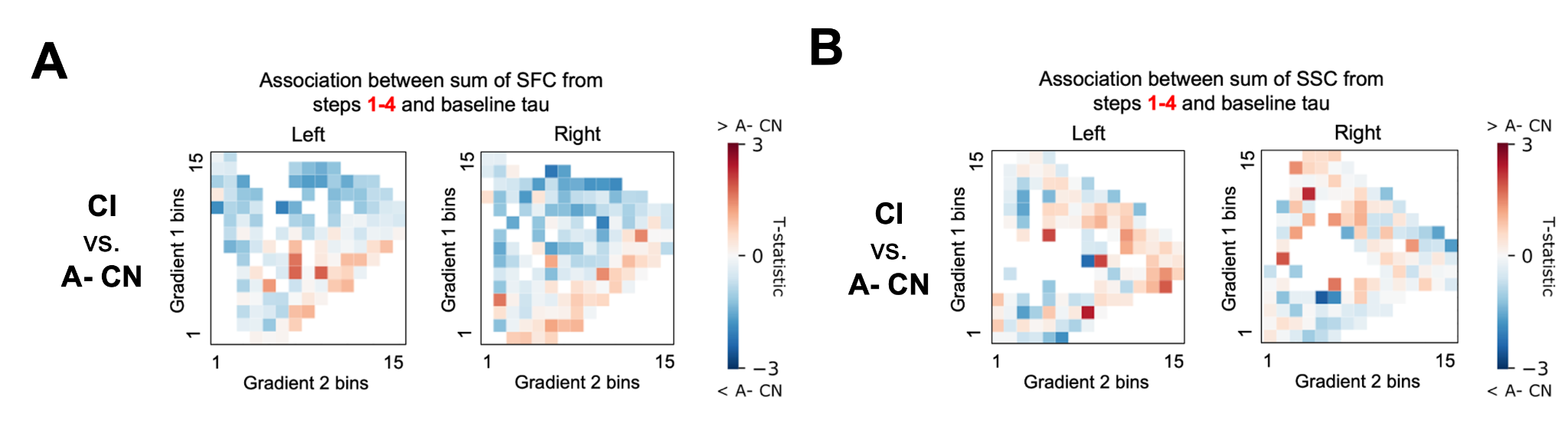


**Supplementary Figure 11:** **(A)** T-statistic of the linear regression between each ROI’s baseline tau and the ROI-wise difference between the sum of SFC summed across steps 1-4 for CI (N=75) and the A- CN (N=102) group average for the left and right hemispheres. ​​See Fig. 4 for this analysis repeated using all steps 1-7. **(B)** T-statistic of the linear regression between each ROI’s baseline tau and the ROI-wise difference between the sum of SSC summed across steps 1-4 for CI (N=75) and the A- CN (N=102) group average for the left and right hemispheres. See Fig. 5 for this analysis repeated using all steps 1-7. Color scale indicates t-statistic. Red pixels indicate increased SSC in A+ CN/CI compared to A- CN, while blue pixels indicate reduced SSC in A+ CN/CI compared to A- CN. CN: cognitively normal; CI: cognitively impaired; ROI: region-of-interest; SFC: stepwise functional connectivity; SSC: stepwise structural connectivity.
